# IFNγ binding to extracellular matrix prevents fatal systemic toxicity

**DOI:** 10.1038/s41590-023-01420-5

**Published:** 2023-02-02

**Authors:** Josephine Kemna, Evelyne Gout, Leon Daniau, Jessica Lao, Kristoffer Weißert, Sandra Ammann, Ralf Kühn, Matthias Richter, Christine Molenda, Anje Sporbert, Dario Zocholl, Robert Klopfleisch, Anja Schütz, Hugues Lortat-Jacob, Peter Aichele, Thomas Kammertoens, Thomas Blankenstein

**Affiliations:** 1 Max-Delbrück Center for Molecular Medicine in the Helmholtz Association, Molecular Immunology and Gene Therapy, Berlin, Germany; 2 Institut de Biologie Structurale, UMR 5075, University Grenoble Alpes, Centre National de la Recherche Scientifique, Commissariat à l’Énergie Atomique et aux Énergies Alternatives, Grenoble, France; 3 Institute for Immunodeficiency, Medical Center—University of Freiburg, Faculty of Medicine, University of Freiburg, Freiburg, Germany; 4 Faculty of Biology, Albert-Ludwigs-University of Freiburg, Freiburg, Germany; 5 Center for Chronic Immunodeficiency (CCI), Medical Center—University of Freiburg, Faculty of Medicine, University of Freiburg, Freiburg, Germany; 6 Transgenic Core Facility, Max-Delbrück Center for Molecular Medicine in the Helmholtz Association, Berlin, Germany; 7 Advanced Light Microscopy Core Facility, Max-Delbrück Center for Molecular Medicine in the Helmholtz Association, Berlin, Germany; 8 Charité—Universitätsmedizin Berlin, Corporate Member of Freie Universität Berlin and Humboldt-Universität zu Berlin, Institute of Biometry and Clinical Epidemiology, Berlin, Germany; 9 Department of Veterinary Medicine, Institute of Veterinary Pathology, Freie Universität Berlin, Berlin, Germany; 10 Protein Production & Characterization Core Facility, Max-Delbrück Center for Molecular Medicine in the Helmholtz Association, Berlin, Germany; 11 Institute of Immunology, Charité Unversitätsmedizin, Campus Buch, Berlin, Germany

**Keywords:** Interferons, Chronic inflammation

## Abstract

Interferon-γ (IFNγ) is an important mediator of cellular immune responses, but high systemic levels of this cytokine are associated with immunopathology. IFNγ binds to its receptor (IFNγR) and to extracellular matrix (ECM) via four positively charged C-terminal amino acids (KRKR), the ECM-binding domain (EBD). Across evolution, IFNγ is not well conserved, but the EBD is highly conserved, suggesting a critical function. Here, we show that IFNγ lacking the EBD (IFNγ^ΔKRKR^) does not bind to ECM but still binds to the IFNγR and retains bioactivity. Overexpression of IFNγ^ΔKRKR^ in tumors reduced local ECM binding, increased systemic levels and induced sickness behavior, weight loss and toxicity. To analyze the function of the EBD during infection, we generated IFNγ^ΔKRKR^ mice lacking the EBD by using CRISPR–Cas9. Infection with lymphocytic choriomeningitis virus resulted in higher systemic IFNγ^ΔKRKR^ levels, enhanced sickness behavior, weight loss and fatal toxicity. We conclude that local retention of IFNγ is a pivotal mechanism to protect the organism from systemic toxicity during prolonged immune stimulation.

## Abstract (web summary)

Here, the authors show that IFNγ binding to heparan sulfate is a mechanism to restrain IFNγ at the site of production, thereby preventing high systemic levels of this cytokine and associated immunopathology.

## Main

Throughout evolution, the immune system has evolved increasingly powerful weapons against pathogens. The price of the arms race between the immune system and pathogens is the risk of overshooting immune responses and subsequent immunopathology^1^. Therefore, counteracting mechanisms, such as regulatory T cells or immune checkpoints that restrict effector T cells, have evolved, which diminish effector function^2–4^. Arguably, cytokines, such as interferon-γ (IFNγ), are the most toxic components of the immune response if they are released systemically in large amounts and for extended periods of time^5,6^. Infections usually occur locally, and IFNγ is secreted locally by T cells after recognition of antigen-presenting target cells and can spread around 800 µm, the equivalent of 30–40 cell layers^7,8^.

**Fig. 1:**
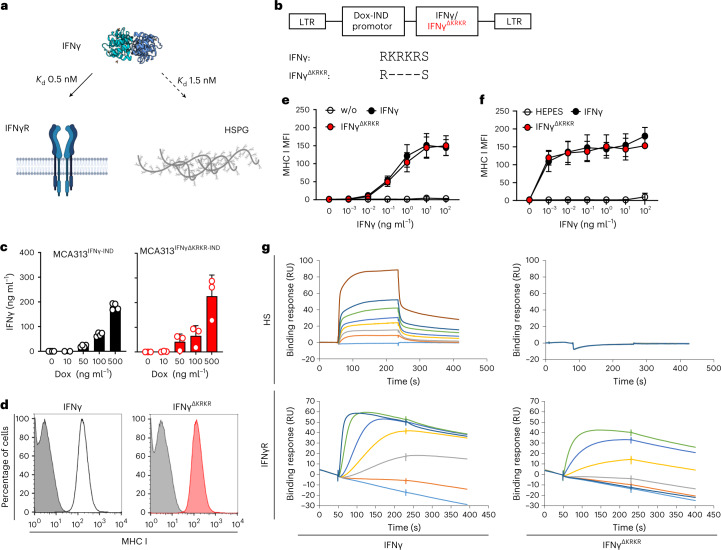
The evolutionarily conserved KRKR motif of IFNγ is necessary for binding to HS. **a**, IFNγ binds to the IFNγR and HSPG. **b**, Construct flanked by long terminal repeats (LTR) for generation of MCA313 cancer cells with Dox-inducible (Dox-IND) expression of different IFNγ variants (MCA313^IFNγ-IND^ and MCA313^IFNγΔKRKR-IND^). **c**, Dox-dependent expression of IFNγ variants in MCA313 cells in vitro. The data show the means + s.d. of *n* = 4 (MCA313^IFNγ-IND^) and *n* = 3 (MCA313^IFNγΔKRKR-IND^) experiments. **d**, Upregulation of MHC class I (H-2K^b^/H-2D^b^) molecules on B16-F10 cells after treatment with 1 ng ml^–1^ IFNγ (left) or IFNγ^ΔKRKR^ (right). B16-F10 cells were cultured for 48 h with supernatants from MCA313 (gray), MCA313^IFNγ-IND^ (black line) or MCA313^IFNγΔKRKR-IND^ (red histogram) cells, respectively, and analyzed by flow cytometry. Shown is one representative experiment out of four. **e**, IFNγ or IFNγ^ΔKRKR^ dose-dependent upregulation of MHC class I on B16-F10 cells. Shown are means ± s.d. of four experiments; MFI, mean fluorescence intensity. **f**, Biological activity of recombinant (*E. coli*-produced) IFNγ and IFNγ^ΔKRKR^ variants as indicated and analyzed in **e**. Shown are means ± s.d. of two individual biological experiments. **g**, IFNγ (left) or IFNγ^ΔKRKR^ (right) was injected over an HS-activated surface (top) or an IFNγR1-activated surface (bottom) over 180 s, and the binding response in resonance units (RU) was recorded as a function of time. Each set of sensorgrams was obtained with IFNγ at (from bottom to top) 0, 25, 50, 75, 100, 150, 200 and 500 nM for the HS surface and at 0, 1, 2.5, 5, 10, 25 and 50 nM for the IFNγR1 surface. Source data

In addition to binding to the ubiquitously expressed IFNγ receptor (IFNγR) with an affinity (*K*_d_) of 0.5 nM, IFNγ also binds to the heparan sulfate (HS) moiety of the extracellular matrix (ECM) with an affinity of 1.5 nM (Fig. 1a)^9^. Binding to the ECM is mediated by four positively charged amino acids (KRKR) at the C terminus of IFNγ. The biological role of IFNγ binding to the ECM is unknown. On the basis of cell culture experiments, different hypotheses were developed. One hypothesis is that the C-terminal part of IFNγ (amino acids 95–133 containing the KRKR motif) is essential for biological activity^10^. It was also suggested that the KRKR motif acts as a nuclear localization signal^11^ or facilitates binding to the IFNγR^12^. Because of the strong evolutionary conservation of the KRKR motif in the IFNγ protein, we investigated its relevance in in vivo models.

## Results

### The KRKR motif of IFNγ is conserved

Comparing the IFNγ protein sequences between 50 vertebrate species covering 450 million years of evolution showed little conservation and overall low homology (Table 1 and Extended Data Table 1), an observation that is in line with reports showing high species specificity of IFNγ and no conserved ligand–IFNγR interaction motifs^13^. Mouse and human IFNγ, for example, are 41% homologous at the amino acid level and do not cross-react^14^. By contrast, the C-terminal KRKR motif is highly conserved throughout evolution, suggesting an essential biological function (Table 1 and Extended Data Table 1). In several species distantly related to mammals (for example, elephant shark, zebra fish, American bullfrog or alligator), deviations from the KRKR motif were detected. In these cases, amino acids of the KRKR motif were substituted without exception by positively charged amino acids, further suggesting an important evolutionary role of this motif.

**Table 1:** IFNγ sequences across five different taxa

Organism	Motif sequence	Identity to mouse IFNγ (%)	Common name	Taxon
*Mus musculus*	**RKRKRS**	100	Mouse	Mammalia
*Rattus norvegicus*	**RKRKRS**	83	Rat	
*Homo sapiens*	**GKRKRS**	41	Human	
*Ornithorhyncos anatinus*	**RKKRRS**	29	Platypus	
*Taeniopygia guttata*	**YKRKRS**	33	Zebra finch	Aves
*Anas platyrhynchos*	**SKRKRS**	29	Mallard	
*Pelodiscus sinensis*	**NKRKRS**	30	Chinese soft-shelled turtle	Reptilia
*Xenopus tropicalis*	**VKKRKL**	25	Tropical clawed frog	Amphibia
*Lithobates catesbeiana*	**VRRKRG**	22	American bullfrog	
*Oncorhynchus mykiss*	**NRRKRR**	27	Rainbow trout	Bony fish
*Danio rerio*	**ERKRRQ**	22	Zebra fish	
*Callorhinchus milii*	**GRRRRR**	23	Elephant shark	Cartilaginous fish

IFNγ sequences across five different taxa and 450 million years of evolution showing a low degree of homology but high conservation of the KRKR motif at the C terminus. Deviations from the mouse KRKR motif are marked by underlining. The common amino acid single-letter code is shown. All 50 species are shown in Extended Data Table 1.

### IFNγ^ΔKRKR^ loses ECM binding but not IFNγR binding

We were concerned that deletion of the KRKR motif would abolish not only ECM binding but also the biological activity of IFNγ. To test if IFNγ^ΔKRKR^ (lacking the KRKR motif) retained biological activity, we transduced MCA313 fibrosarcoma cells^15^ with retroviruses that allowed for doxycycline (Dox)-induced expression of either IFNγ or IFNγ^ΔKRKR^ (Fig. 1b). Both cytokines were inducibly expressed in similar amounts (Fig. 1c). Additionally, IFNγ and IFNγ^ΔKRKR^ induced upregulation of H-2K^b^/H-2D^b^ molecules on B16-F10 melanoma cells in a similar and concentration-dependent manner (Fig. 1d,e).

We expressed and purified recombinant IFNγ and IFNγ^ΔKRKR^ in *Escherichia coli*, and both cytokines upregulated the expression of major histocompatibility complex class I (MHC class I) on B16-F10 cells (Fig. 1f). We then analyzed the interactions of IFNγ and IFNγ^ΔKRKR^ with IFNγR1, the ligand-binding chain of the heterodimeric IFNγR and HS by surface plasmon resonance (SPR). Binding of IFNγ and IFNγ^ΔKRKR^ to an IFNγR1 surface or to an HS surface was measured at various ligand concentrations. Deletion of the ECM-binding domain (EBD; IFNγ^ΔKRKR^) decreased the on rate of the binding response to IFNγR1 (Fig. 1g), in agreement with previous studies demonstrating that the basic C-terminal residues of the cytokine enhance the on rate of IFNγ binding to IFNγR1 (ref. ^12^) but do not prevent the formation of the complex nor its stability. By contrast, deletion of the EBD completely abrogated binding to HS (Fig. 1g), which was also supported by previous nuclear magnetic resonance-based studies showing that IFNγ amino acids experiencing chemical shift variation after binding to HS are exclusively localized in the basic motif of the cytokine C terminus^16^. Thus, IFNγ binds to both IFNγR1 and HS, whereas the IFNγ^ΔKRKR^ variant retains IFNγR binding and biological activity but loses ECM binding.

### Reduced colocalization of IFNγ^ΔKRKR^–GFP and ECM

**Fig. 2:**
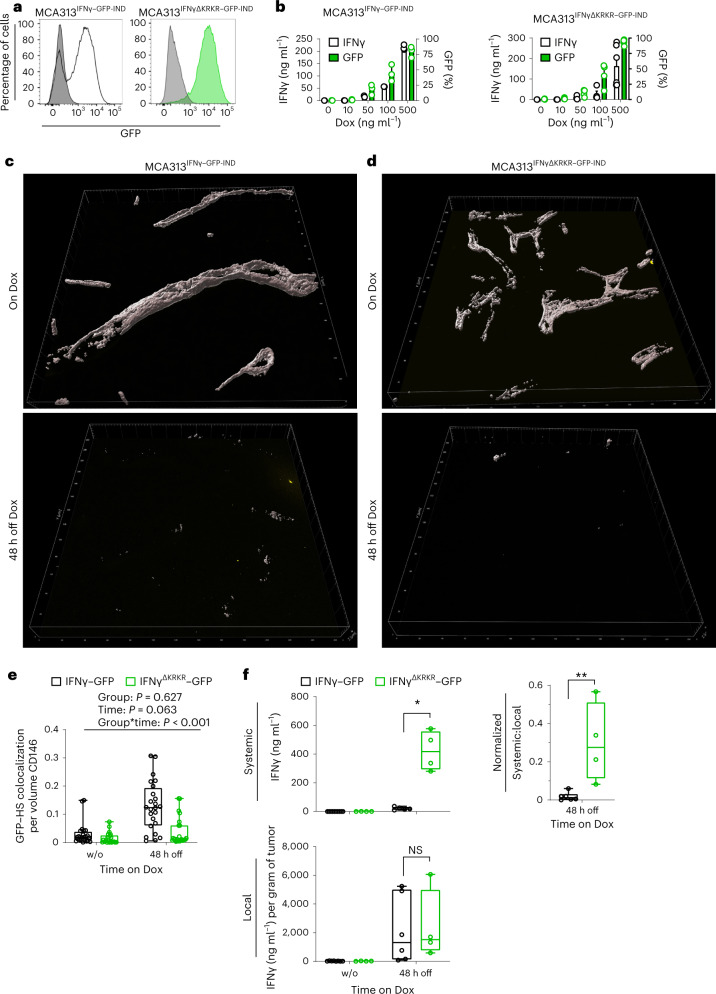
IFNγ colocalizes with HS in vivo and is retained locally. **a**, Representative examples of inducible expression of IFNγ–GFP (left, black line) and IFNγ^ΔKRKR^–GFP (right, green histogram) in MCA313 cells after stimulation with 500 ng ml^–1^ Dox. Gray histograms show absence of expression in the absence of Dox. **b**, Dox-dependent expression of IFNγ–GFP and IFNγ^ΔKRKR^–GFP in MCA313 cells in vitro. Data are shown as mean and s.d. of *n* = 2–4 (MCA313^IFNγ–GFP-IND^) and *n* = 4–6 (MCA313^IFNγΔKRKR–GFP-IND^) experiments. **c**,**d**, *Ifng*^–/–^*Ifngr1*^–/–^ mice were injected with either MCA313^IFNγ–GFP-IND^ (**c**) or MCA313^IFNγΔKRKR–GFP-IND^ cells (**d**). Dox was administered via the drinking water (on Dox) when tumors reached 200–300 mm^3^. Tumors were induced for a minimum of 3 d, and Dox was withdrawn (48 h off Dox). Three-dimensional surface reconstructions of the colocalization channel of 16-µm sections acquired by confocal microscopy from tumor tissue are shown. Using Imaris, colocalization channels were calculated from colocalizing voxels of GFP and HS within the CD146 volume. Shown are representative volumes from on Dox and 48 h after Dox withdrawal (48 h off). Representative stainings of the same specimens are depicted in Extended Data Fig. 1c,d. Whole 3D *z* stacks are also in Supplementary Video 1. **e**, Colocalization as determined by normalizing the calculated colocalization volume for GFP and HS to the CD146 volume. Five to six confocal *z* stacks were acquired from four mice per group. Volumes were calculated using Imaris 9.7.2. **f**, Serum levels (systemic) and tumor levels (local) of IFNγ–GFP variants in *Ifng*^–/–^*Ifngr1*^–/–^ mice were determined by ELISA 48 h after Dox withdrawal. Mice without Dox served as controls (w/o). The fraction of the respective IFNγ–GFP variant systemically available was determined by normalizing systemic levels to local levels. Data from *n* = 4–10 mice per group from two independent experiments are shown, and data from individual mice are plotted. Median values are shown as the center lines, minima are the lower limits, maxima are the upper limits, and quartiles are the bounds of boxes. Statistical analyses were performed using the Mann–Whitney test (two sided) or a linear mixed model with random intercept for each individual and an interaction effect between time and group (**e**); NS, not significant; *P = 0.0159 and **P = 0.0095. Source data

**Extended Data Fig. 1:**
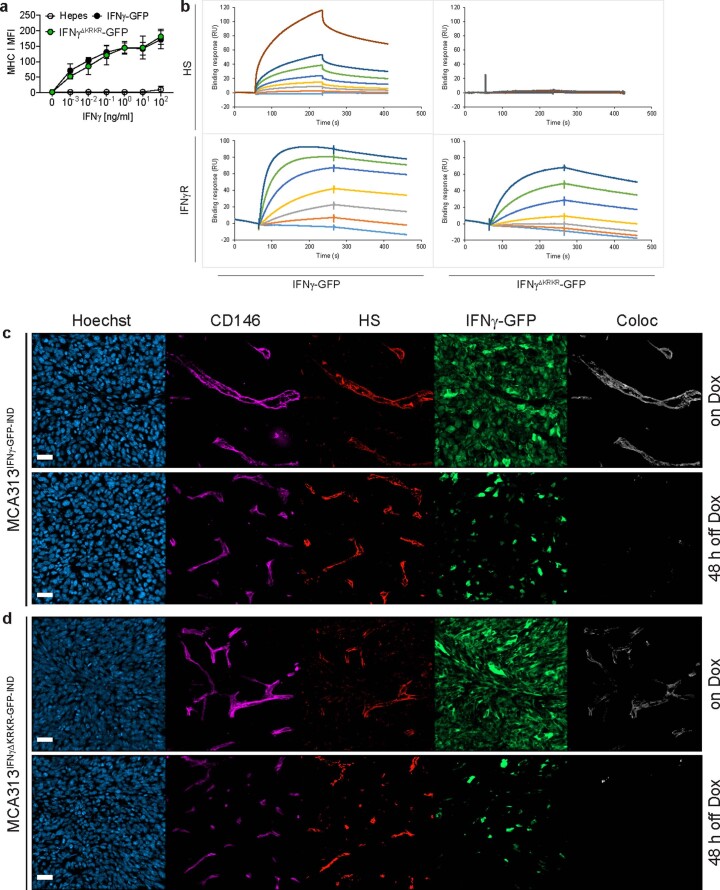
IFNγ^ΔKRKR^-GFP binds to the IFNγR, but not HS. **a**, Upregulation of MHC I on B16-F10 cells by different recombinantly produced IFNγ-GFP variants. B16-F10 cells were cultured with the indicated IFNγ variant for 48 h or Hepes as control. Shown are means ± s.d. of two individual biological experiments. **b**, IFNγ^ΔKRKR^-GFP binds to immobilized IFNγR1, but binding to HS is abrogated. IFNγ-GFP (upper and lower left panel) or IFNγ^ΔKRKR^-GFP (upper and lower right panel) were injected over a HS-activated surface (upper row) or a IFNγR1-activated surface (lower row) during 180 seconds, and the binding response in resonance units (RU) was recorded as a function of time. Each set of sensorgrams was obtained with IFNγ-GFP at (from bottom to top): 0, 25, 50, 75, 100, 150, 200 and 500 nM for the HS surface and: 0, 1, 2.5, 5, 10, 25 and 50 nM for the IFNγR1 surface. **c, d**, IFNγ^-/-^IFNγR^-/-^ mice were injected with either MCA313^IFNγ-GFP-IND^ (c) or MCA313^IFNγΔKRKR-GFP-IND^ cells (d). Dox was administered via the drinking water (on Dox) when tumors reached 200-300 mm³. Tumors were induced for a minimum of 3 days and then Dox was withdrawn (48 h off Dox). Tumor tissue was stained for CD146, HS and nuclei (Hoechst). Representative confocal images from 2 experiments per time point are shown. Scale bars indicate 30 µm. Related data of the same samples are shown in Fig. 2c, d and Extended Data Movie 1.

Direct evidence that the KRKR motif mediates ECM binding in vivo is lacking; thus, we analyzed whether IFNγ and HS colocalize in vivo. For IFNγ visualization in tissue, IFNγ–green fluorescent protein (GFP) fusion proteins were used because detection of IFNγ using antibodies can be misleading^17^, and, of note, we wished to detect extracellular, HS-bound IFNγ. Recombinant fusion proteins IFNγ–GFP and IFNγ^ΔKRKR^–GFP were similarly bioactive in upregulating MHC class I expression on B16-F10 cells (Extended Data Fig. 1a). As measured by SPR, IFNγ^ΔKRKR^–GFP had retained IFNγR binding but lost HS binding, while IFNγ–GFP bound to both IFNγR and HS (Extended Data Fig. 1b). MCA313 cells were generated and secreted similar amounts of IFNγ–GFP (MCA313^IFNγ–GFP-IND^) or IFNγ^ΔKRKR^–GFP (MCA313^IFNγΔKRKR–GFP-IND^) in a Dox-inducible manner (Fig. 2a,b). Tumors were established in *Ifng*^–/–^/*Ifngr1*^–/–^ mice, which prevented IFNγR binding and excluded competition with endogenous IFNγ for HS binding. Expression of IFNγ–GFP and IFNγ^ΔKRKR^–GFP was induced by Dox for a minimum of 3 d when tumors reached 200–300 mm^3^. Then, Dox was withdrawn for 48 h to stop cytokine production, and tumor tissues were analyzed for colocalization between IFNγ–GFP and HS. Tumor sections were stained with antibodies specific to CD146 and HS proteoglycan (HSPG) to visualize endothelial cells and HS (Extended Data Fig. 1c,d). The HSPG antibody (clone A7L6) binds to perlecan domain IV, a major HSPG constituent of the basement membrane in blood vessels^18^. This way, we could exclude residual GFP signal from the cancer cells. Immunohistochemical analysis of tumor tissue revealed tenfold more colocalization of IFNγ–GFP voxels with HS voxels than colocalization of IFNγ^ΔKRKR^–GFP voxels with HS voxels within the CD146 volume (Fig. 2c–e and Supplementary Video 1). Serum IFNγ^ΔKRKR^–GFP levels were significantly higher than serum IFNγ–GFP levels 48 h after Dox withdrawal (Fig. 2f). Local concentrations were not different between IFNγ–GFP and IFNγ^ΔKRKR^–GFP; however, we surmise that the ECM-bound IFNγ–GFP is less quantitatively extracted from the tumor tissue than IFNγ^ΔKRKR^–GFP. The normalized systemic IFNγ:local IFNγ values from the same animals were significantly higher for IFNγ^ΔKRKR^–GFP than for IFNγ–GFP (Fig. 2f). We conclude that the KRKR motif mediates binding to HS in vivo and acts like a sponge to retain IFNγ locally, thereby reducing its systemic availability.

### Local IFNγ^ΔKRKR^ expression causes severe systemic toxicity

**Fig. 3:**
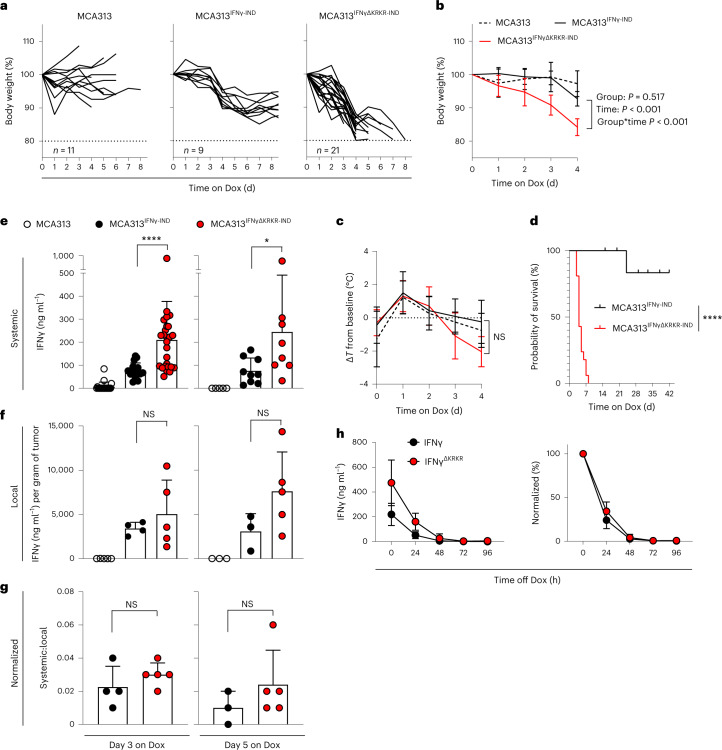
Locally produced IFNγ^ΔKRKR^ induces wasting disease and increases serum levels in tumor-bearing mice. **a**, Mice bearing MCA313, MCA313^IFNγ-IND^ or MCA313^IFNγΔKRKR-IND^ tumors received Dox in the drinking water starting at a tumor volume of 487 ± 110 mm^3^, 487 ± 95 mm^3^ and 516 ± 119 mm^3^, respectively, and weight loss was analyzed over time. Cumulative data from four experiments are shown. **b**, Mean (±s.d.) weight loss in mice from **a**. **c**, Body temperature (shown as mean ± s.d.) of four mice per group from **a** was monitored. **d**, Survival of mice in **a**. Mice reaching maximal tumor volume or end of experiment are censored. **e**, IFNγ in the sera of mice on day 3 (left, MCA313^IFNγ-IND^ *n* = 16 and MCA313^IFNγΔKRKR-IND^ *n* = 26) and day 5 (right, MCA313^IFNγ-IND^ *n* = 9 and MCA313^IFNγΔKRKR-IND^ *n* = 8) for the mice depicted in **a**. **f**, Local concentrations of IFNγ on day 3 at the tumor site were determined for MCA313^IFNγ-IND^ (*n* = 4) and MCA313^IFNγΔKRKR-IND^ tumor-bearing mice (*n* = 5). On day 5, local IFNγ concentrations were determined for MCA313^IFNγ-IND^ tumor-bearing mice (*n* = 3) and for MCA313^IFNγΔKRKR-IND^ tumor-bearing mice (*n* = 5). **g**, Ratios of systemic to local IFNγ levels were calculated for mice with both values available. On day 3, the systemic:local ratio in MCA313^IFNγ-IND^ tumor-bearing mice (*n* = 4) was 0.023 ± 0.013 and was 0.030 ± 0.007 in MCA313^IFNγΔKRKR-IND^ tumor-bearing mice (*n* = 5). On day 5, the ratio was calculated to be 0.010 ± 0.010 for MCA313^IFNγ-IND^ tumor-bearing mice (*n* = 3) and 0.024 ± 0.021 for MCA313^IFNγΔKRKR-IND^ tumor-bearing mice (*n* = 5). All values given are mean ± s.d. **h**, Serum kinetics of different IFNγ variants in *Ifng*^–/–^*Ifngr1*^–/–^ mice bearing the respective MCA313 tumors. Mice received Dox for 3 d when tumors reached 200–400 mm^3^ (MCA313^IFNγ-IND^ *n* = 6, MCA313^IFNγΔKRKR-IND^ *n* = 6), Dox was withdrawn, and IFNγ serum levels were analyzed over time. Data from mice from two independent experiments are shown as mean ± s.d. Significance was calculated using the Mantel–Cox test for survival, a Mann–Whitney test (two sided) was used for **e**–**g**, and a linear mixed model with random intercept for each individual and an interaction effect between time and group was used for **b** and **c**; *P = 0.036 and ****P ≤ 0.0001. Source data

**Extended Data Fig. 2:**
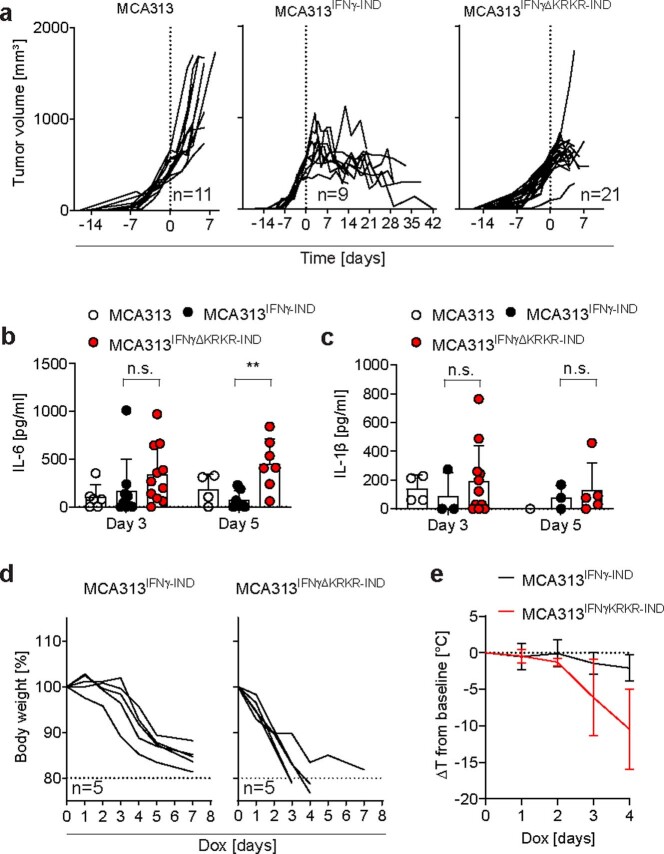
Locally produced IFNγ^ΔKRKR^ increases serum levels of IL-1β and IL-6 and causes systemic toxicity in the absence of T cells. **a**, C57Bl/6 mice harboring MCA313, MCA313^IFNγ-IND^ or MCA313^IFNγΔKRKR-IND^ tumors received Dox in the drinking water starting at a tumor volume of 487 ± 110 mm³, 487 ± 95 mm³ and 516 ± 119 mm³, respectively, as indicated by the dashed vertical line. **b**, IL-6 in the serum of mice on day 3 (left, MCA313 (white circles, n = 6), MCA313^IFNγ-IND^ (black circles, n = 9) or MCA313^IFNγΔKRKR-IND^ tumors (red circles, n = 11)) and day 5 (right, MCA313 (white circles, n = 4), MCA313^IFNγ-IND^ (black circles, n = 7) or MCA313^IFNγΔKRKR-IND^ tumors (red circles, n = 7, p = 0,0041)). Individual mice, mean and s.d. are shown. **c**, IL-1β in the serum of mice on day 3 (left, MCA313 (white circles, n = 4), MCA313^IFNγ-IND^ (black circles, n = 3) or MCA313^IFNγΔKRKR-IND^ (red circles, n = 11)) and day 5 (right, MCA313 (white circles, n = 1), MCA313^IFNγ-IND^ (black circles, n = 3) or MCA313^IFNγΔKRKR-IND^ tumors (red circles, n = 5)). Individual mice, mean and s.d. are shown. **d**, **e**, Rag2-deficient mice bearing either MCA313^IFNγ-IND^ or MCA313^IFNγΔKRKR-IND^ tumors received Dox in the drinking water starting at a tumor volume of 539 ± 98 mm³ and 449 ± 98 mm³, respectively. **d**, weight loss as well as **e**, body temperature (mean ± s.d.) was monitored for one week after Dox induction. Data from 1 experiment with n = 5 mice per group is shown. Significance was calculated for **b** and **c**, using the Mann-Whitney test (two-sided) with n.s. not significant, ** p ≤ 0.01, and for **d**, differences between MCA313^IFNγ-IND^ and MCA313^IFNγΔKRKR-IND^ tumor bearing mice were analyzed between days 1 and 4 after Dox induction using a linear mixed effects model revealing a strong and increasing difference in weight (p = 0.006 on day 1, p < 0.001 on days 2-4, p-values are adjusted for multiple comparisons by Holm method). Source data

We investigated how deletion of the EBD affects biological functions and health status in IFNγR-competent C57BL/6N mice by local IFNγ or IFNγ^ΔKRKR^ release from solid tumors. In mice with established MCA313, MCA313^IFNγ-IND^ or MCA313^IFNγΔKRKR-IND^ tumors, the IFNγ variants were induced by Dox administration via the drinking water. The growth of MCA313^IFNγ-IND^ and MCA313^IFNγΔKRKR-IND^ tumors slowed, while MCA313 tumors progressively grew (Extended Data Fig. 2a). Within a few days, the release of IFNγ^ΔKRKR^ induced sickness-related behavior, as observed by reduced movement and curled body posture. IFNγ^ΔKRKR^ induced severe weight loss (around 20%; Fig. 3a,b) and reduced the body temperature (–2 °C Δ*T*) within 4 d (Fig. 3c), and the mice had to be killed due to a predefined humane endpoint (Fig. 3d). By contrast, induction of IFNγ induced mild weight loss and little change in body temperature, and mice recovered after several days (Fig. 3a–d). While differences in weight loss between both groups were statistically significant, sickness was accompanied by a wider range of temperatures (that is, higher standard deviation) so that the differences of the latter did not reach statistical significance. Severe systemic toxicity induced by IFNγ^ΔKRKR^ correlated with increased serum levels of the proinflammatory cytokines interleukin-1β (IL-1β) and IL-6 (Extended Data Fig. 2b,c), which, however, again did not reach statistical significance. Local IFNγ and IFNγ^ΔKRKR^ concentrations in the tumor were not different. We believe that the ECM-bound IFNγ could not be extracted from the tumor tissue as efficiently as free IFNγ (Fig. 3f). Serum levels of IFNγ after Dox induction were increased about threefold on days 3 and 5 in mice harboring MCA313^IFNγΔKRKR-IND^ tumors compared to in mice harboring MCA313^IFNγ-IND^ tumors (Fig. 3e); however, the normalized ratio of systemic to local IFNγ^ΔKRKR^ was increased compared to IFNγ (Fig. 3g). To exclude the possibility that the increased systemic levels of IFNγ^ΔKRKR^ compared to IFNγ were due to altered serum half-life of IFNγ^ΔKRKR^, both cytokines were induced in *Ifng*^–/–^/*Ifngr1*^–/–^ mice bearing MCA313^IFNγ-IND^ or MCA313^IFNγΔKRKR-IND^ tumors. After Dox withdrawal, serum levels of IFNγ and IFNγ^ΔKRKR^ were analyzed over time. There was no difference in the serum half-life of both proteins (Fig. 3h). Importantly, severe systemic toxicity was also observed when IFNγ^ΔKRKR^, but not IFNγ, was induced in tumors in T cell- and B cell-deficient *Rag2*^–/–^ mice (Extended Data Fig. 2d,e), suggesting that toxicity was not mediated by T cells. In summary, the deletion of the EBD of IFNγ leads to fatal immunopathology caused by local expression in the tumor tissue.

### Fatal toxicity in lymphocytic choriomeningitis virus (LCMV)-infected IFNγ^ΔKRKR^ mice

**Fig. 4:**
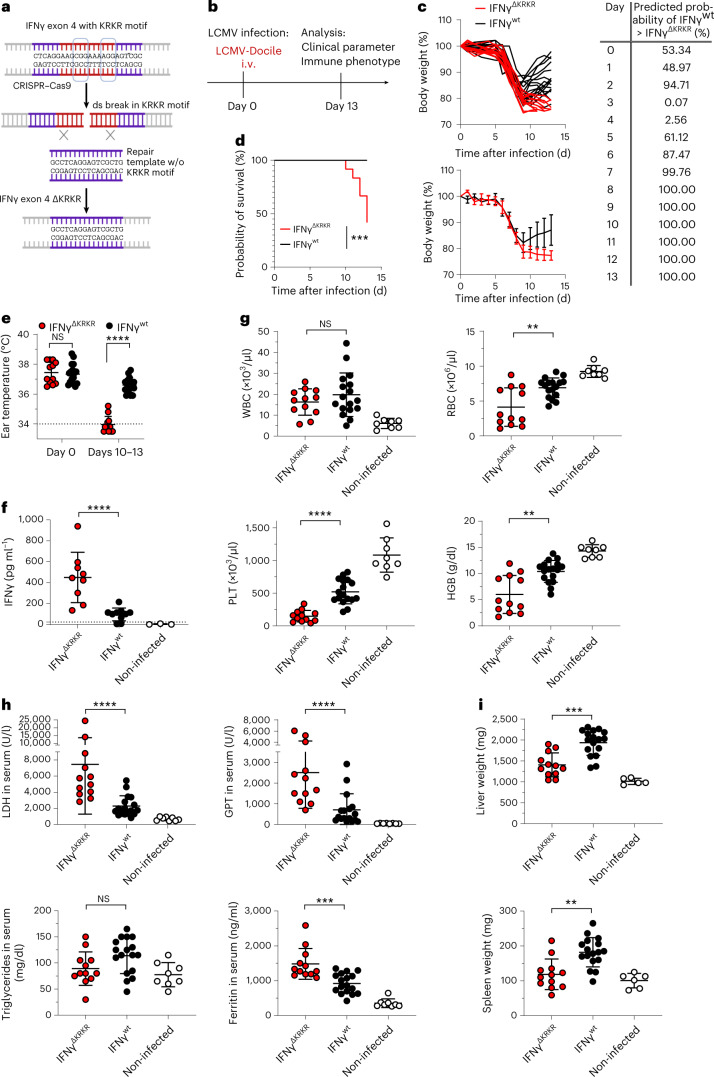
IFNγ^ΔKRKR^ mice succumb to viral infections. **a**, CRISPR–Cas9 approach for the generation of IFNγ^ΔKRKR^ mice; ds, double-strand. **b**, Scheme of IFNγ^ΔKRKR^ (red) or IFNγ^wt^ (black) mice infected with LCMV-Docile and monitored for 13 d; i.v., intravenous. **c**, Weight curves of IFNγ^ΔKRKR^ (red) and IFNγ^wt^ (black) mice following infection. IFNγ^ΔKRKR^ mice deteriorated from days 8 to 9 as determined by weight loss, while IFNγ^wt^ mice recovered. The table shows the predicted probability of the weight of IFNγ^wt^ mice being greater than the weight of IFNγ^ΔKRKR^ mice in percent. **d**, Survival analysis of mice shown in **c** (*P* = 0.0003). Mice from the IFNγ^ΔKRKR^ group had to be killed due to increased weight loss starting from day 10. **e**, Ear temperature of IFNγ^ΔKRKR^ and IFNγ^wt^ mice before and 10–13 d after LCMV infection. **f**, Serum IFNγ levels in IFNγ^ΔKRKR^ and IFNγ^wt^ mice at endpoint analysis. Non-infected mice are shown as controls. **g**, Parameters from the blood analysis of IFNγ^ΔKRKR^ mice show reduced white blood cell (WBC), red blood cell (RBC; *P* = 0.0077) and platelet (PLT) counts as well as reduced hemoglobin (HGB; *P* = 0.0018) compared to IFNγ^wt^ mice after infection. Non-infected mice are shown as controls. **h**, Elevated LDH, GPT and ferritin (*P* = 0.0003) levels but similar amounts of triglycerides were detected in the sera of IFNγ^ΔKRKR^ mice (red) compared to IFNγ^wt^ mice (black). **i**, Liver and spleen pathology in IFNγ^ΔKRKR^ mice (red) is indicated by reduced organ weight (*P* = 0.0002 for liver weight and *P* = 0.001 for splenic weight) after infection compared to IFNγ^wt^ mice (black). Shown are data from 9–17 mice from three to four independent experiments, and data are shown as mean ± s.d. Significance was calculated by Mantel–Cox test for survival or Mann–Whitney test (two sided); **P ≤ 0.01, ***P ≤ 0.001 and ****P ≤ 0.0001. For the data in **c**, statistical analyses were performed using a linear mixed model with random intercept for each individual and an interaction effect between time and group, where time was parameterized by restricted cubic splines with four knots to account for non-linearity. From this model, the predictive distribution was computed via bootstrap samples, and the predicted probability of IFNγ^wt^ > IFNγ^ΔKRKR^ was calculated. Source data

**Extended Data Fig. 3:**
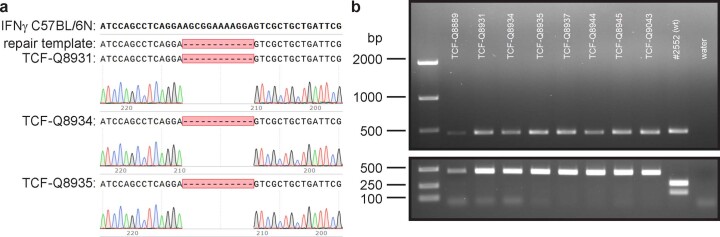
Generation of IFNγ^ΔKRKR^ mice. **a**, A PCR fragment of exon 4 of IFNγ from CRISPR/Cas9-modified founder mice was sequenced. Sequences were aligned to the native IFNγ sequence (C57BL/6 N, NC_000076.6) and the repair template to validate deletion events. Three out of nine mice are shown. **b**, The deletion of an AciI restriction site within the KRKR motif indicates zygosity in founder mice. Upper panel shows PCR product, lower panel AciI digestion. The 480 bp fragment indicates homozygous deletion of 12 bp encoding the KRKR motiv. Each lane corresponds to one founder, each founder was genotyped at least twice. The C57BL/6 N mouse #2552 was used as a wild type control (wt).

**Extended Data Fig. 4:**
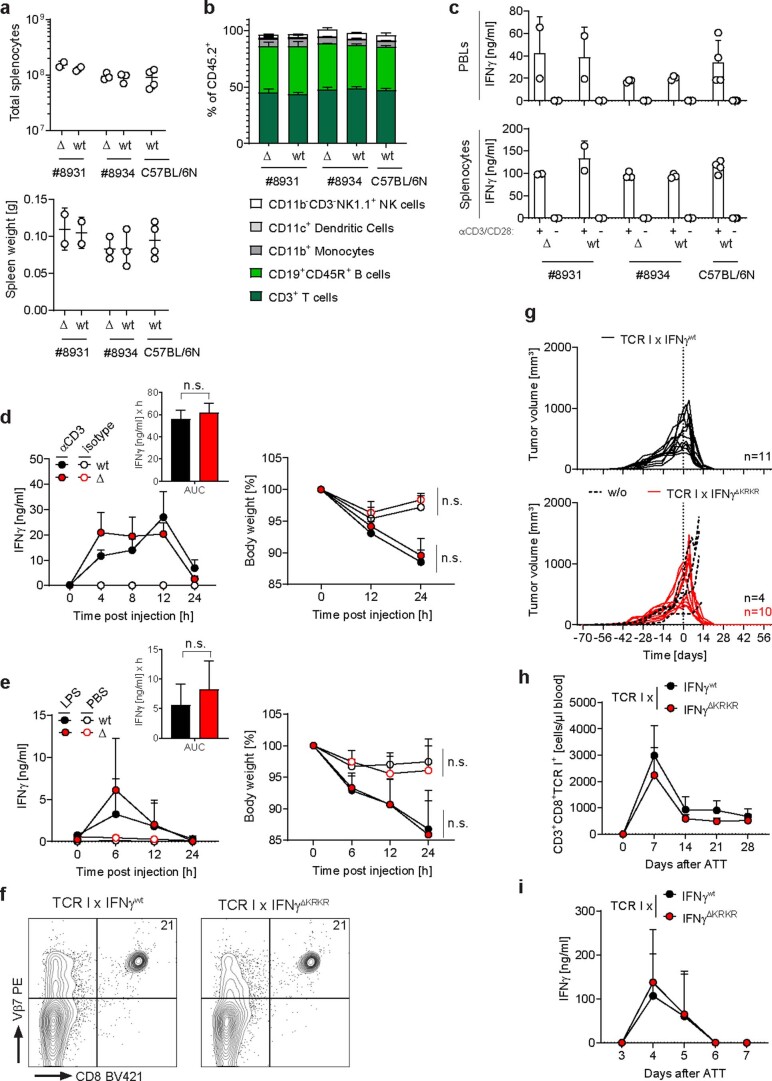
Anti-CD3 antibody treatment, LPS treatment and tumor rejection induce transient IFNγ production and no immunopathology in IFNγ^ΔKRKR^ mice. **a** Total splenocytes, splenic weight, and **b** splenocyte populations as determined by flow cytometry in IFNγ^ΔKRKR^ (Δ/Δ), IFNγ^WT^ (wt/wt) control littermates of two IFNγ^ΔKRKR^ mouse lines and C57Bl6/N mice. IFNγ^ΔKRKR^ mice from 1 and C57BL/6 N mice from 2 experiments are shown. Individual mice, mean and s.d. are shown. **c**, IFNγ secretion by CD3/CD28 stimulation of PBL (upper panel) and splenocytes (lower panel) of IFNγ^ΔKRKR^ and IFNγ^wt^ control littermate of two IFNγ^ΔKRKR^ lines and C57BL/6 N mice. Individual IFNγ^ΔKRKR^ mice from 1 and individual C57BL/6 N mice from 2 experiments are shown. **d**, Serum IFNγ levels (left panel) and relative body weight (right panel) in IFNγ^ΔKRKR^ (n = 5, red circles) and wild type control littermates (IFNγ^wt^, n = 5, black circles) after i.v. administration of 20 µg anti-CD3 antibodies. Control mice (IFNγ^ΔKRKR^ n = 3, IFNγ ^wt^ n = 3) received isotype antibodies. Shown are mean and s.d.. Inlay shows Area under Curve (AUC ± SEM) of serum IFNγ level through the observed time to depict total secreted IFNγ over 24 h. **e**, Serum IFNγ level (left panel, mean and s.d.) and AUC ± SEM of serum level (inlay) and relative body weight (right panel, mean and s.d.) in IFNγ^ΔKRKR^ (n = 7, red circles) and wild type control littermates (IFNγ^wt^, n = 3, black circles) after i.p. administration of 5 µg/g body weight of LPS. Control mice (IFNγ^ΔKRKR^ n = 4, IFNγ^wt^ n = 2) received PBS i.v. IFNγ^ΔKRKR^ mice from 2 experiments (control littermates only one experiment) are shown. **f**, Representative flow cytometry plots from two experiments of splenocytes from TCR-I x IFNγ^ΔKRKR^ mice, analyzed on the day of transfer. Splenocytes were stained for CD8 and Vβ7 antibodies to determine proportion of CD8^+^Vβ7^+^ T cells per spleen. **g**, Rag1^-/-^ mice were treated with 1-2 ×10^6^ TCR-I^+^ splenocytes from TCR-I x IFNγ^wt^ donor mice (black lines, n = 11) or TCR-I x IFNγ^ΔKRKR^ donor mice (red lines, right, n = 10) or left untreated (dashed lines, right, n = 4) when 16.113 tumors reached 500 mm³ in size, indicated by the dashed vertical line. Tumor size was monitored for up to 60 days after transfer. **h**, Numbers of CD3^+^CD8^+^TCR-I^+^IFNγ^wt^ (black circles) and CD3^+^CD8^+^TCR-I^+^IFNγ^ΔKRKR^ (red circles) T cells in the blood of mice in g were analysed by flow cytometry over time (shown as mean and s.d.). **i**, Serum from mice in g was collected daily from day 3 to day 7 after T cell transfer. IFNγ serum levels are shown as mean and s.d.. Significance was calculated using unpaired two-sided t-test for AUC analysis (d,e) with n.s. not significant. For weight in d,e, significance was calculated by contrast tests following a linear mixed model with random intercept for each individual and an interaction effect between time and group. For tumor volume, a linear mixed model was fitted with random intercept for each individual and an interaction effect between time and group, where time was parameterized by restricted cubic splines with 5 knots to account for non-linearity, and from this model the predictive distribution was computed via bootstrap samples and the predicted probability of IFNγ^ΔKRKR^ > IFNγ^wt^ was calculated. Source data

To analyze the role of the IFNγ KRKR motif in a virus infection model, we generated a mouse line with the KRKR motif deleted in the endogenous *Ifng* gene by using CRISPR–Cas9 technology. Two guide RNAs (gRNAs), a template lacking the KRKR sequence and CRISPR–Cas9 protein were electroporated into C57BL/6N zygotes to introduce double-strand breaks and homology-directed repair, followed by transfer into pseudopregnant mice (Fig. 4a). After sequence analysis, 8 of 31 pups born were identified to have a precise deletion of the KRKR coding sequence on both alleles, shown for three founders (Extended Data Fig. 3a). Additionally, the deletion of an AciI restriction site in the KRKR coding sequence was used to assess zygosity of the eight founder mice (Extended Data Fig. 3b). IFNγ^ΔKRKR^ mice showed no gross abnormalities and developed and bred normally. Histopathology did not identify pathologic changes or signs of neoplasia, degeneration or inflammation in IFNγ^ΔKRKR^ mice compared to wild-type control littermates (IFNγ^wt^). Spleen tissue and blood were analyzed to assess the distribution of immune cell populations. No differences in total splenocytes between IFNγ^ΔKRKR^ and IFNγ^wt^ mice were detected (Extended Data Fig. 4a). Populations of T cells, B cells, monocytes, dendritic cells and natural killer cells were detected with similar frequencies in IFNγ^ΔKRKR^ and IFNγ^wt^ mice (Extended Data Fig. 4b). IFNγ production after in vitro stimulation of peripheral blood lymphocytes or splenocytes with antibodies to CD3/CD28 was also similar (Extended Data Fig. 4c).

We tested IFNγ^ΔKRKR^ mice with three types of experimental stimulation known to result in a rapid increase in IFNγ followed by a similarly rapid decrease to baseline: (1) injection of anti-CD3 (ref. ^19^), (2) injection of lipopolysaccharide (LPS)^20^ or (3) rejection of solid tumors^15^. In both IFNγ^ΔKRKR^ and IFNγ^wt^ mice, treatment with anti-CD3 and LPS induced similar rapid increases in IFNγ serum levels and transient weight loss, followed by decreases to baseline levels within 24 h (Extended Data Fig. 4d,e). *Rag1*^–/–^ mice bearing large established SV40 large T antigen-expressing tumors^21^ were then treated with CD8^+^ T cells transgenic for a T cell antigen receptor (TCR) specific for peptide I of the large T antigen (TCR-I) expressing either IFNγ^ΔKRKR^ or IFNγ^wt^ (Extended Data Fig. 4f). In this model, successful T cell therapy requires IFNγ as an effector molecule^21^. IFNγ^ΔKRKR^ and IFNγ^wt^ TCR-I T cells similarly expanded after transfer and eradicated the tumor with similar rejection kinetics (maximal predicted probability of 63% for a group difference at any time; Extended Data Fig. 4g,h). In mice treated with IFNγ^ΔKRKR^ or IFNγ^wt^ TCR-I T cells, IFNγ serum levels with similar kinetics peaked on day 4 and dropped to baseline on day 6 (Extended Data Fig. 4i). The data confirm that IFNγ^ΔKRKR^ has retained biological activity and that ECM binding of IFNγ is not necessary for tumor rejection in vivo. Furthermore, in models of transient IFNγ production, the EBD is not necessary to prevent immunopathology.

**Extended Data Fig. 5:**
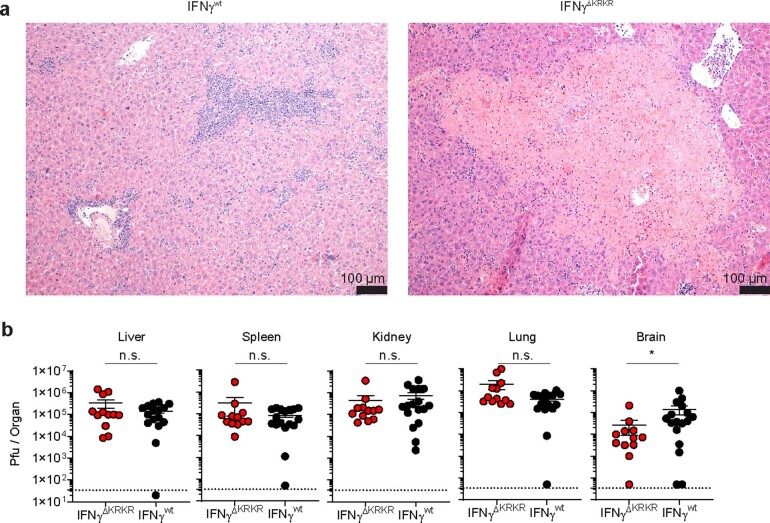
Liver necrosis in IFNγ^ΔKRKR^ mice and similar virus titers in organs of LCMV-infected IFNγ^ΔKRKR^ and IFNγ^wt^ mice. Histopathological analysis of organs (liver, kidney, brain, spleen and lung) was performed at the endpoint of LCMV infection experiments (day 10 to 13). **a**, Shown are H&E from liver tissue. Analysis of two independent experiments revealed that 0/5 wildtype littermates and 2/6 IFNγ^ΔKRKR^ mice showed massive liver necrosis. **b**, Virus titers in organs of IFNγ^ΔKRKR^ mice (n = 12) and IFNγ^wt^ control littermate (n = 17) were determined between day 10-13. LCMV-DOCILE was detected in all organs analysed and no meaningful difference was observed between IFNγ^ΔKRKR^ and IFNγ^wt^ mice. Combined results from four experiments (mean ± s.e.m.) are shown. Significance was calculated using the two-sided Mann-Whitney test with n.s. not significant, * p = 0.038.

**Extended Data Fig. 6:**
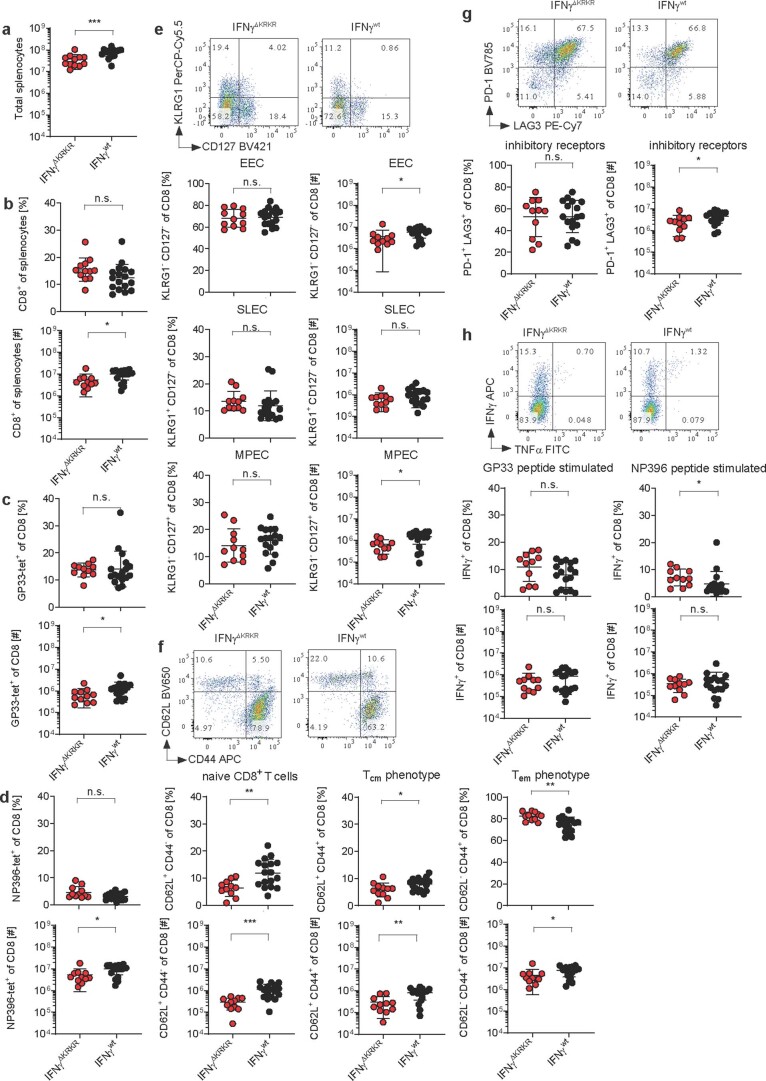
Phenotype of CD8 T cells in IFNγ^∆KRKR^ mice after LCMV infection. IFNγ^∆KRKR^ and IFNγ^wt^ mice were infected i.v. with LCMV-Docile and analysed between day 10-13. **a**, splenocyte counts and **b**, CD8 T cells within splenocytes. **c**, GP33-specific cells within CD8 T cells. **d**, NP396-specific cells within CD8 T cells. **e**, Differentiation of effector CD8 T cells according to their expression of KLRG-1 and CD127 (exemplary staining shown in the upper two panels) into “EECs” (early effector cells), “SLECs” (short lived effector cells) and “MPECs” (memory precursor effector cells) was followed. **f**, Based on CD62L and CD44 expression (exemplary staining shown in the upper two panels), naïve CD8 T cells were discriminated from effector cells with a T_cm_ (central memory) or T_em_ (effector memory) phenotype. **g**, PD-1 and LAG3 expression (exemplary staining shown in the upper two panels) of CD8 T cells after in vitro restimulation with GP33 or NP396 peptides. **h**, IFNγ expression (exemplary staining shown in the upper two panels) of CD8 T cells after in vitro restimulation with GP33 or NP396 peptides. In b-h, always relative frequencies (%) as well as absolute cell numbers (#) are shown as individual mice, mean and s.d.. IFNγ^ΔKRKR^ (n = 11) and IFNγ^wt^ (n = 17) mice from four independent experiments are shown. Significance in a-h was calculated using the Mann-Whitney test (two-sided) with n.s. not significant, * p ≤ 0.05, ** p ≤ 0.01, *** p ≤ 0.001.

We hypothesized that the high evolutionary selective pressure for the KRKR motif of IFNγ is associated with chronic antigen stimulation, as occurs during infections with delayed pathogen elimination. To test this hypothesis, we infected IFNγ^ΔKRKR^ and IFNγ^wt^ mice with intermediate doses of LCMV-Docile (Fig. 4b), which induces a self-limiting infection with prolonged immune stimulation due to delayed virus clearance^2^. Starting around 6 d after infection, both IFNγ^wt^ and IFNγ^ΔKRKR^ mice lost body weight (Fig. 4c). While IFNγ^wt^ mice regained weight and recovered from day 9 onward, IFNγ^ΔKRKR^ mice continued to lose weight, and 7 of 12 mice had to be killed until day 13 according to endpoint criteria (Fig. 4d). IFNγ^ΔKRKR^ mice showed strongly reduced peripheral body temperature (Fig. 4e) and increased serum IFNγ levels (Fig. 4f). Severe sickness behavior and immunopathology in IFNγ^ΔKRKR^ mice was evidenced by decreased red blood cell and platelet counts as well as decreased hemoglobin compared to IFNγ^wt^ mice, while white blood cell counts were comparable (Fig. 4g). Compared to IFNγ^wt^ mice, IFNγ^ΔKRKR^ mice showed strongly increased lactate dehydrogenase (LDH), glutamate–pyruvate transaminase (GPT) and ferritin levels but normal serum triglyceride levels (Fig. 4h). As assessed by histology, some LCMV-infected IFNγ^ΔKRKR^, but not IFNγ^wt^, mice showed areas of necrosis in the liver (Extended Data Fig. 5a), indicating more severe liver damage in IFNγ^ΔKRKR^ mice. Enhanced toxicity also correlated with reduced liver and spleen weights in IFNγ^ΔKRKR^ mice (Fig. 4i). No meaningful differences in viral load in liver, spleen, kidney, lung and brain were detected between IFNγ^wt^ and IFNγ^ΔKRKR^ mice (Extended Data Fig. 5b). In line with the less pronounced splenomegaly, significantly lower spleen cell counts were observed in IFNγ^∆KRKR^ mice than in IFNγ^wt^ mice (Extended Data Fig. 6a). No differences between the two mouse groups were observed in the percentages of CD8^+^ T cells and the LCMV-specific GP33- or NP396-reactive CD8^+^ T cells, while absolute numbers of LCMV-specific GP33- or NP396-reactive CD8^+^ T cells were slightly decreased in IFNγ^ΔKRKR^ mice (Extended Data Fig. 6b–d). The relative sizes of effector subpopulations, namely early effector cells (KLRG-1^–^/CD127^–^), short-lived effector cells (KLRG-1^+^/CD127^–^) and memory precursor effectors cells (KLRG-1^–^/CD127^+^), were not affected by IFNγ lacking the EBD, but again, absolute numbers of early effector cells and memory precursor effector cells were slightly decreased in IFNγ^ΔKRKR^ mice (Extended Data Fig. 6e). IFNγ^∆KRKR^ mice exhibited lower percentages and absolute numbers of naive CD8^+^ T cells and higher percentages but lower absolute numbers of effector memory T cells. Also, lower relative and absolute numbers of CD8^+^ T cells with a central memory T cell phenotype were observed in IFNγ^∆KRKR^ mice (Extended Data Fig. 6f). Comparable coexpression of inhibitory receptors PD-1 and LAG-3 on CD8^+^ T cells was observed, but again, reduced numbers were detectable in IFNγ^ΔKRKR^ mice (Extended Data Fig. 6g). The percentage of IFNγ-expressing CD8^+^ T cells was comparable after in vitro restimulation with GP33 peptide and was slightly increased in NP396 peptide-stimulated CD8^+^ T cells from IFNγ^∆KRKR^ mice; no differences in absolute numbers between both groups were observed (Extended Data Fig. 6h). In summary, differentiation of effector CD8^+^ T cells into various subpopulations was largely comparable between IFNγ^∆KRKR^ and IFNγ^wt^ mice in the context of LCMV infection with slightly lower absolute numbers of some CD8^+^ T cell subpopulations observed in IFNγ^∆KRKR^ mice.

**Extended Data Fig. 7:**
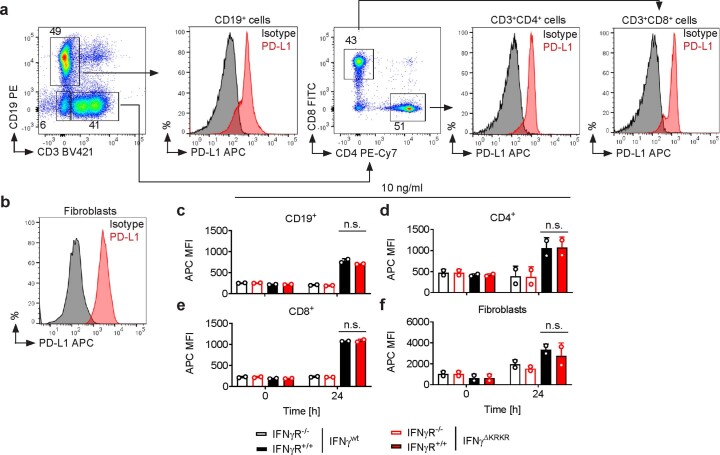
Similar upregulation of PD-L1 by IFNγ and IFNγ^ΔKRKR^. Splenocytes or fibroblasts were cultured in the presence of 10 ng/ml IFNγ or IFNγ^ΔKRKR^. Cells were analyzed 24 h after cytokine exposure for PD-L1 expression. **a**, Gating strategy for splenocytes, and **b**, Example staining of fibroblasts cultured with IFNγ. **c**, CD19; **d**, CD4; **e**, CD8; and **f**, fibroblasts are shown. Bar diagrams show MFI of PD-L1 staining from two independent biological experiments (mean ± s.d.). Comparing average MFI of cells cultured for 24 h with either IFNγ or IFNγ^ΔKRKR^ using an unpaired t-test revealed no significant differences (n.s.).

**Extended Data Fig. 8:**
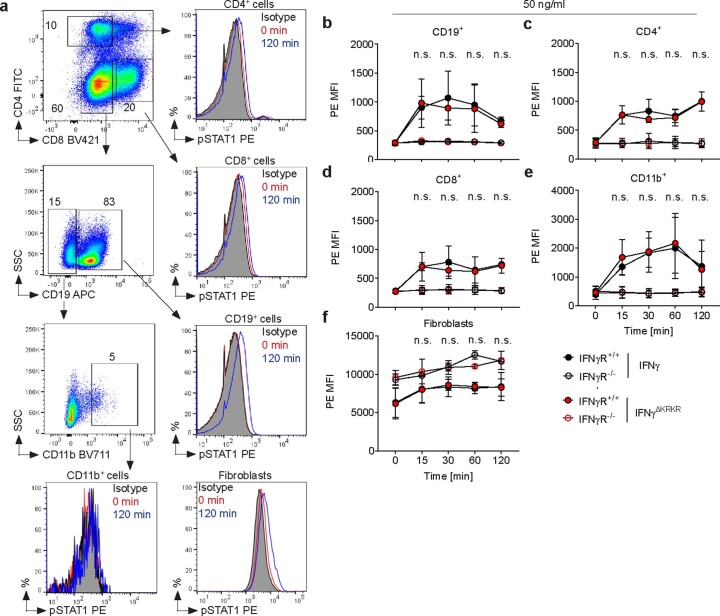
Similar induction of Stat1 by IFNγ- and IFNγ^ΔKRKR^. Splenocytes or fibroblasts were cultured in 50 ng/ml IFNγ or IFNγ^ΔKRKR^. **a**, Splenocytes or fibroblasts were analyzed at different time points after cytokine exposure for phospho-Stat1 induction in vitro by flow cytometry. **b**, CD19; **c**, CD4; **d**, CD8; **e**, CD11b; and **f**, fibroblasts are shown. Curve graphs show kinetics of MFI changes of anti-phospho-Stat1 staining. Shown are mean ± s.d. values from 2 (b,c,d,f) or 3 (e) independent biological experiments. Comparing average MFI of cells cultured with either IFNγ or IFNγ^ΔKRKR^ using a paired two-sided t-test revealed no significant (n.s.) differences for any of the time points analyzed (15, 30, 60, 120 min).

To exclude the possibility that the slight decrease in IFNγ^∆KRKR^ in the on rate of the binding response to IFNγR1 (detected in the SPR analysis (Fig. 1g)) altered receptor-proximal signaling, as has been described for other mutant IFNγ molecules^22^, and thus would have caused the toxicity, we compared signaling properties of both IFNγ^wt^ and IFNγ^∆KRKR^ in terms of PD-L1 upregulation and STAT1 phosphorylation. Splenocytes and fibroblasts incubated with IFNγ^wt^ and IFNγ^∆KRKR^ upregulated the expression of PD-L1 in a similar fashion (Extended Data Fig. 7) and with similar kinetics and magnitude induced the phosphorylation of STAT1 (Extended Data Fig. 8) in IFNγR-competent, but not IFNγR-deficient, cells. This suggested that the immunopathology was not due to alterations in IFNγR signaling or PD-L1 regulation by IFNγ^∆KRKR^. Because T cell responses and viral load were largely similar and receptor-proximal signaling was not altered by deletion of the EBD, we conclude that the severe clinical phenotype was likely associated with elevated serum IFNγ^∆KRKR^ levels, as was also observed in the reductionist tumor model.

## Discussion

We have shown that IFNγ binds to HSPG of the ECM in vivo in close proximity to its production and is locally retained for at least 48 h. Binding is mediated by four positively charged amino acids at the C terminus of IFNγ interacting with the negatively charged HS. This motif, which in most species is KRKR, is often flanked by additional positively charged amino acids, for example, RKRKRR in the koala. In 50 species covering 450 million years of vertebrate evolution, the motif consisting of four positively charged amino acids is conserved. Deviations from the KRKR motif are without exception positively charged amino acids, for example, RRRR in the elephant shark. In human IFNγ, the motif occurs twice in the C terminus (KRKR … RGRR), and in mice, the motif occurs only once (KRKR). Therefore, investigating mouse IFNγ was straightforward in this hypothesis-driven approach based on the assumption that evolutionary conservation is a strong indicator for its importance.

The ability of IFNγ to bind to HS is well recognized^9^. Five hundred and eighty proteins bind to HS/heparin, and numerous cytokines, chemokines or growth factors contain a motif of several, not necessarily contiguous, positively charged amino acids^23^. It was suggested that the binding of various proteins to HS plays multiple roles, such as in the regulation of leukocyte development, leukocyte migration, immune activation and inflammatory processes^24^. Most analyses to date were based on in vitro assays. It has been suggested that cell surface HS facilitates binding of IFNγ to IFNγR^12^ or that the KRKR motif acts as a nuclear localization signal, enhancing the biological activity of IFNγ^11^. As IFNγ^ΔKRKR^ has retained normal biological activity (for example, IFNγR-proximal signaling), these mechanisms appear of minor importance. It was also suggested that local IFNγ binding to HS increases its availability^25^ or that binding of IFNγ to tumor phosphatidylserine converts transient exposure into long-lived inflammation^26^. However, IFNγ^ΔKRKR^ T cells rejected large tumors as efficiently as IFNγ^wt^ T cells, indicating that local retention of IFNγ is not required in a model of IFNγ-dependent rejection of established solid tumors. Whether binding of IFNγ^ΔKRKR^ to phosphatidylserine is retained and plays a role in tumor rejection remains to be determined. Additionally, the pharmacokinetics of IFNγ have been analyzed, for example, when bound to heparin, and binding of IFNγ to HS may protect the cytokine from degradation and regulate its activity^27,28^. However, IFNγ–HS binding has not been implicated as a mechanism to prevent immunopathology, as shown here in two in vivo models in a side-by-side comparison of IFNγ^wt^ and IFNγ^ΔKRKR^. In the first model, the local expression of IFNγ^ΔKRKR^ compared to IFNγ^wt^ in solid tumors resulted in reduced local retention, increased systemic serum levels and wasting disease within a few days. Similarly, in the second model, IFNγ^∆KRKR^, but not IFNγ^wt^, mice experienced severe immunopathology with increased systemic IFNγ^∆KRKR^ levels following LCMV infection. Sequestration seems to be particularly important during infection, with delayed virus clearance and prolonged IFNγ production.

Several mechanisms that reduce immunopathology have been described. Regulatory T cells as well as immune checkpoints diminish T cell effector function^2–4^. Pathogens can be tolerated to minimize tissue damage^29^. Finally, previous pathogen exposure can increase the kinetics and magnitude of immune responses to subsequent, even antigen-unrelated, infections, thereby minimizing the risk of immunopathology^30,31^. Mice in the current study were kept under specific pathogen-free (SPF) conditions; therefore, future analysis of mice with a natural, wild microbiome will be of interest^32^. Together, we describe an evolutionarily conserved mechanism to prevent immunopathology by restraining IFNγ at its local site of production and avoiding IFNγ toxicity from systemic release at high concentrations.

## Methods

### Sequence homology analysis

IFNγ protein sequences of different species were compared using FASTA identifiers (Supplementary Table 1) and UniProtKB (https://www.uniprot.org/uniprot/). Sequence homology to mouse was determined using sequence alignment via BLAST (accessed on 30 August 2021; https://blast.ncbi.nlm.nih.gov/Blast.cgi?PAGE=Proteins).

### Recombinant IFNγ variants and SPR

Proteins used for binding studies (Supplementary Table 2) were expressed in *E. coli*. cDNA sequences without the leader peptide were codon optimized and cloned into a pET28-N-His_6_-SUMO vector. IFNγ^ΔKRKR^ and IFNγ^ΔKRKR^–GFP mutants were generated using the QuikChange site-directed mutagenesis kit (Stratagene). The extracellular domain (amino acids 26–254) of the IFNγR1 protein was cloned based on mouse cDNA into a pET26b-N-His_6_-SUMO vector, resulting in periplasmic expression of an N-terminal His_6_-SUMO-tagged protein bearing an additional C-terminal His_6_ tag. All proteins were produced using T7 express competent *E. coli* (New England Biolabs) cotransformed with the pRARE plasmid. For purification of IFNγ proteins, bacteria were collected and resuspended in lysis buffer (1× PBS (pH 7.4), 0.2 M NaCl, 5% glycerol supplemented with cOmplete EDTA-free protease inhibitor cocktail (Roche), 0.25% (wt/vol) 3-((3-cholamidopropyl)-dimethylammonio)-1-propanesulfonate, 1 mM phenylmethylsulfonyl fluoride, 100 µl of lysozyme (100 mg ml^–1^) and 1 µl of benzonase (250 U µl^–1^; Merck)) and lysed by two freeze–thaw cycles. Proteins were purified on a HisTrap FF crude column (Cytavia), followed by size-exclusion chromatography on a Superdex 75 column (XK 26 × 60, Cytavia) or Superdex 200 column (XK 26 × 60, Cytavia) for C-terminal GFP fusion proteins, respectively. The N-terminal His_6_-SUMO tag was cleaved with yeast Ulp1p SUMO protease (produced in-house) followed by a gel filtration step and reapplication of the cleaved protein on the Ni^2+^ affinity column. For purification of IFNγR1, bacteria were lysed by osmotic shock using 0.2 M Tris-HCl (pH 8.0), 0.5 mM EDTA (pH 8.0), 0.5 M sucrose and 1 mM phenylmethylsulfonyl fluoride as lysis buffer. Purification, as described above, was followed by cleavage of the N-terminal His_6_-SUMO tag and a final gel filtration step on a 10/300 Superdex 200 GL increase column (Cytavia). Recombinant proteins were stored in 20 mM HEPES (pH 7.5) and 0.2 M NaCl at –80 °C until further use. Binding kinetics of IFNγ variants to HS and the recombinant IFNγR1 were determined by SPR on a Biacore T200 (GE Healthcare) using a dextran Series S Sensor Chip CM4 (GE Healthcare). For analysis of HS binding, commercial HS derived from porcine intestinal mucosa has been used (Celsus Laboratories), and this preparation was previously characterized^33^. The average molecular weight of HS was determined to be 12 kDa with a polydispersity of 1.59, and its sulfation degree, evaluated by S and N elemental analysis, was ~1.4 sulfate groups per disaccharide, on average. For IFNγR1, the Sensor Chip was coated with anti-His_5_ (Qiagen), onto which the recombinant IFNγR1 proteins were immobilized.

### pMOV1-T2 vectors for Dox-inducible IFNγ variant expression

The pMOV1-T2 vector was used for inducible IFNγ expression^15^. Variants with deletion of the KRKR motif (ΔKRKR) were generated with the QuikChange Lightning site-directed mutagenesis kit (Agilent) according to manufacturer’s instructions. To generate the pMOV1-T2-IFNγ^ΔKRKR^^-IND^ vector, the primers 5′-ttgccggaatccagcctcaggagtcgctgctgagcgg-3′ and 5′-tcagcagcgactcctgaggctggattccgg-3′ were used to introduce the 12-base pair (bp) deletion. For deletion in the pMOV1-T2-IFNγ^ΔKRKR-GFP-IND^ vector, the primers 5′-gagagcagcctgaggagccgctgcggcggaggcg-3′ and 5′-cgcctccgccgcagcggctcctcaggctgctctcggg-3′ were used.

### Cell lines

HEK293T cells were cultured in DMEM (GlutaMAX, Gibco, Life Technologies) supplemented with 10% fetal calf serum (heat inactivated; Pan Biotech) and 1× antibiotic–antimycotic (Gibco, Life Technologies). MCA313 is a methylcholanthrene-induced fibrosarcoma cell line derived from IFNγR1-deficient mice^15^, B16-F10 is a melanoma cell line^34^, and 16.113 is a SV40 large T antigen-expressing carcinoma cell line^35^. The latter cells were cultured in RPMI 1640 medium (Gibco, Life Technologies) supplemented with 5% fetal calf serum, 1× non-essential amino acids, 1 mM sodium pyruvate and 1× antibiotic–antimycotic (Gibco, Life Technologies).

### Transduction, cloning and characterization of MCA313 cells

MCA313 cells were transduced using a retroviral vector. Vector particles were produced in HEK293T cells. Transduced cells were cultured, and single cells were sorted to establish cell lines. IFNγ variant expression was analyzed by seeding 4 × 10^5^ cells in 2 ml per well in a six-well plate and culturing them without or with 10–500 ng ml^–1^ Dox for 48 h. IFNγ concentration in the supernatant was assessed by enzyme-linked immunosorbent assay (ELISA; BD Biosciences). MCA313 cells harboring IFNγ–GFP fusion proteins were analyzed for GFP expression using a MACSQuant 10 (Miltenyi) and FlowJo 10 (BD Biosciences).

### Bioactivity of IFNγ variants

The bioactivity of IFNγ variants was tested by analyzing MHC class I upregulation on B16-F10 cells. B16-F10 cells (5 × 10^5^) were incubated in RPMI supplemented with supernatants from cells producing the IFNγ variants or with proteins produced in *E. coli*. Concentrations of IFNγ proteins were determined by ELISA and added to B16-F10 cell cultures in concentrations from 0.001 to 100 ng ml^–1^. After 48 h, B16-F10 cells were stained for MHC class I expression using biotinylated anti-H-2K^b^/H-2D^b^ (clone 28-8-6) followed by APC-streptavidin (both BD Biosciences).

To further analyze both IFNγ and IFNγ^ΔKRKR^, primary splenocytes or immortalized fibroblasts from IFNγR1-deficient or WT C57BL/6 mice were incubated with supernatants from cells producing the two variants. PD-L1 expression and intracellular STAT1 phosphorylation were analyzed by flow cytometry. Cells were acquired on a FACSymphonyA1 and analyzed using FlowJo. For PD-L1 analysis, 1 × 10^6^ splenocytes or 5 × 10^4^ fibroblasts were incubated in the presence of 10 ng ml^–1^ IFNγ or IFNγ^ΔKRKR^, and after 0 h (untreated) and 24 h, cells were stained with anti-PD-L1 for 30 min (splenocytes were additionally stained for CD4, CD8 and CD19) and analyzed by flow cytometry.

For analysis of STAT1 phosphorylation, the same cells (splenocytes or immortalized fibroblasts) and supernatants were used. Cells were incubated with 50 ng ml^–1^ IFNγ or IFNγ^ΔKRKR^ and fixed at 37 °C for 15 min with Fixation Buffer (Biolegend) according to the protocol for intracellular staining of phosphoproteins using True-Phos Perm buffer (Biolegend). Thereafter, cells were permeabilized using True-Phos Perm buffer at –20 °C overnight and stained for 30 min in Cell Staining buffer.

### Mice

Mouse experiments were approved by Landesamt für Gesundheit und Soziales Berlin (G-322/10, G-58/16 and G-114/17) and Regierungspräsidium Freiburg (G-15/168). In tumor transplantation experiments, the maximum tumor volume allowed was 15 × 15 × 15 mm. This size was never exceeded in the experiments. Mice were randomly assigned into groups when injected with tumor cells. For adoptive T cell therapy (ATT) experiments, mice were allocated to groups based on equal distribution in tumor size between different groups. Investigators were not blinded, as endpoint criteria of mouse experiments were defined before experiments. No data were excluded from analysis. Data sets were acquired prospectively and analyzed in a retrospective manner. Animals used were group housed with two to five mice in individually ventilated cages (Tecniplast, Green Line or Blue Line) and maintained under identical housing conditions, such as a 12-h light/12-h dark cycle (light cycle 6:30 to 18:30), standard pelleted mouse diet (ssniff, article number v1124-300) ad libitum, free access to water, 22 ± 2 °C room temperature and a relative humidity of 55 ± 10%. The cages contained wooden bedding material (Tapvei Estonia, Aspen bedding, 4HK, 10 kg), nestlets (ssniff, H3279-10), a red plastic house (ZOONLAB) and paper tunnels (ZOONLAB, 3084030) as cage enrichment. Animals were handled by male and female caretakers and technicians.

*Rag1*^–/–^ (B6.129S7-*Rag1*^*tm1Mom*^/J, 002216), *Rag2*^–/–^ (B6.Cg-*Rag2*^*tm1.1Cgn*^/J, 008449), TCR-I (B6.Cg-Tg(TcraY1,TcrbY1)416Tev/J, 005236), C57BL/6N (005304), C57BL/6J (000664), B6.129S7-*Ifng*^*tm1Ts*^/J (002287) and B6.129S7-*Ifngr1*^*tm1Agt*^/J (003288) mouse strains were obtained from The Jackson Laboratory. All mice were bred and housed under SPF conditions at the animal facility of the Max-Delbrück-Center. *Ifng*^–/–^*Ifngr1*^–/–^ mice were obtained by crossing B6.129S7-*Ifng*^*tm1Ts*^/J (002287) and B6.129S7-*Ifngr1*^*tm1Agt*^/J (003288) mice. *Ifng*^–/–^*Ifngr1*^–/–^ mice were genotyped by PCR. *Ifng*^–/–^ primer specific for the mutant allele (5′-CCTTCTATCGCCTTCTTGACG-3′), a primer for the WT allele (5′-AGAAGTAAGTGGAAGGGCCCAGAAG-3′) and a common reverse primer (5′-AGGGAAACTGGGAGAGGAGAAATAT-3′) were used. *Ifngr1*^–/–^ forward primer specific for intron 4 (5′-ATGCAACGGTTTCCACCCCC-3′), a reverse primer specific for the introduced neomycin cassette (5′-CCAGTCATAGCCGAATAGCC-3′) and a reverse primer specific for intron 5 (5′-CCACCTCAGCACTGTCTTCA-3′) were used.

### Antibodies and staining reagents

The antibodies and reagents listed are presented in the following format: (immunogen detected/fluorochrome or conjugate/clone/vendor catalog number/species isotype).

For flow cytometry, the following reagents were used: (CD45.2/APC/104/Biolegend 109814/mouse IgG2a, κ), (CD3/FITC/145-2C11/BD 553062/Armenian hamster IgG1 κ); (CD45.2/BV421/104/Biolegend 109832/mouse IgG2a, κ), (CD19 FITC/6D5/Biolegend 115506/rat IgG2a, κ), (CD11b/BV510/ M1/70/Biolegend 101263/rat IgG2b, κ), (CD11c/FITC/HL3/BD 553801/hamster IgG1), (NK1.1/APC/PK136/Miltenyi 130-117-379/mouse IgG2a), (H-2K^b^/H-2D^b^-biotin/28-8-6/BD 553575/mouse IgG2a, κ), (staining reagent streptavidin–APC, BD-554067), (CD8/BV421/53-6.7/Biolegend 100753/rat IgG2a, κ), (Vb7/PE/TR310/BD 553216/rat IgG2b, κ), (CD3/APC/145-2C11/Biolegend 100312/Armenian hamster IgG), (CD4/APC-Fire750/RM4-4/Biolegend 116019/rat IgG2b, κ), (CD8/BV510/53-6.7/Biolegend 100751/rat IgG2a, κ), (KLRG-1/PerCP-Cy5.5/2F1/BD 563595/Syrian hamster IgG2, κ), (CD127/BV421/A7R34/Biolegend 135023/rat IgG2a, κ), (CD44/APC/IM7/eBioscience 17-0441-82/rat IgG2b, κ), (CD62L/BV650/MEL-14/Biolegend 104453/rat IgG2a, κ), (PD-1/BV785/29F.1A12/Biolegend 135225/rat IgG2a, κ), (LAG-3/PE-Cy7/C9B7W/Biolegend 125208/rat IgG1 κ), (tetramer custom made: Tet-GP33/PE/Baylor College of Medicine), (tetramer custom made: Tet-NP396/PE/Baylor College of Medicine), (CD45.2/BV711/104/Biolegend 109847/mouse IgG2a, κ), (CD19/PE/6D5/Biolegend 115508/rat IgG2a, κ), (CD4/PE-Cy7/RM4-5/Biolegend 116016/rat IgG2a, κ), (CD8/FITC/53-6.7/Biolegend 100706/rat IgG2a, κ), (CD3/BV421/145-2C11/Biolegend 100336/Armenian hamster IgG), (CD274/APC/10F.9G2/Biolegend 124312/rat IgG2b, κ), (Fc block/93/Biolegend 101302/rat IgG2a, l) and (CD4/FITC/RM4-4/Biolegend 100510/rat IgG2a, κ).

For intracellular cytokine staining, the following reagents were used: (CD8/PerCP-Cy5.5/53-6.7/Biolegend 100733/rat IgG2a, κ), (CD4/BV650/RM4-4/Biolegend 100545/rat IgG2b, κ), (IFNγ/APC/XMG1.2/Biolegend 505810/rat IgG1 κ), (TNF/FITC/MP6-XT22/Biolegend 506304/rat IgG1 κ), (anti-STAT1/PE/4a/BD 612564/mouse IgG2a) and (isotype control/PE/MOPC-173/BD 558595/mouse IgG2a, κ).

For histology, the following regents were used: (HSPG 2/A7L6/Abcam ab2501/rat IgG2a), (goat anti-rat/Alexa 568/Invitrogen A11077), (CD146/Alexa 647/ME-9F1/Biolegend 134702/rat IgG2a, κ) and (staining reagent: Hoechst 33342/Sigma-Aldrich14533).

For in vivo applications, the following reagents were used: (CD3/145-2C11/Biolegend 100340/Armenian hamster IgG1 κ) and (Armenian hamster/Biolegend 400940/IgG1 κ/isotype control).

### Analysis of HS–IFNγ colocalization

To generate samples for histology, 1 × 10^6^ MCA313^IFNγ–GFP-IND^ or MCA313^IFNγΔKRKR–GFP-IND^ cells were injected subcutaneously into the right or left flank of *Ifng*^–/–^*Ifngfr1*^–/–^ mice at 11–43 weeks of age.

Tumor size was measured, and volumes were determined using the ellipsoid volume formula$V = \frac{{\pi \ast a \ast b \ast c}}{6}$. When tumors reached a size of approximately 300 mm^3^ (days 10–12), mice received Dox (1 mg ml^–1^ in 2% glucose) via the drinking water for at least 3 d. Thereafter, Dox was removed. Tumors were excised, and sera were taken. One part was fixed in 4% paraformaldehyde (PFA; Sigma-Aldrich) for 24–48 h at 4 °C, followed by an incubation in 30% sucrose in PBS (Gibco) for 24–48 h at 4 °C. Tissue was mounted in optimum cutting temperature compound (Tissue-Tek, Sakura) and frozen at –80 °C.

For colocalization of IFNγ and HS, 16-µm cryosections of PFA-fixed tissue were stained for HSPG, CD146 and Hoechst. For staining of HS and CD146, antigen retrieval for 6 h at 60 °C with 10 mM citrate buffer (Roth, pH 7.4) was performed. Slides were washed in PBS and blocked in 1% bovine serum albumin (pH 7; Sigma-Aldrich) containing 0.2% Triton X-100 (PanReac AppliChem ITW Reagents) and 0.2% gelatine from coldwater fish (Sigma-Aldrich) for 1 h at room temperature. Slides were washed three times for 5 min each in PBS. HS was visualized using rat anti-HSPG 2 (clone A7L6, Abcam) in antibody diluent (Dako) overnight at 4 °C in the dark. The primary antibody was detected by a goat anti-rat antibody coupled to Alexa 568 (A11077, Invitrogen) incubated for 2 h at room temperature in the dark, followed by simultaneous CD146 and nuclear staining using a directly labeled CD146–Alexa 647 antibody (clone ME-9F1, Biolegend) and Hoechst 33342 (Sigma-Aldrich), incubating for 2 h at room temperature in the dark. Image acquisition and processing of imaging raw data were performed on a Zeiss LSM 980 AiryScan 2 system (Carl Zeiss Microscopy) using the AiryScan MPLX SR-4Y mode with a final pixel size of 0.065 µm × 0.065 µm and a *z* sampling rate of 0.3 µm. Images were acquired with a ×20/0.8-NA Plan-Apochromat Air objective (working distance of 0.55 mm). Diode laser lines at 639 nm (Alexa 647), 561 nm (Alexa 568), 488 nm (GFP) and 405 nm (Hoechst) were used to excite the fluorophores. To detect the fluorophore emissions, detection wavelengths were set to 659–720 nm (Alexa 647), 574–627 nm (Alexa 568), 499–548 nm (GFP) and 422–477 nm (Hoechst). For each section, six separate positions were defined within one region of the sample. Appropriate areas were chosen by abundance of capillaries. For comparability of the samples, all recordings were performed with the same instrument parameter settings. Thresholds for CD146 and HS were set based on negative controls and were applied globally. Only in rare exceptions was the threshold adjusted manually, again using negative controls. For GFP, the threshold was set based on tissue without Dox induction as negative controls to exclude autofluorescence from the tissue. Sections with necrotic or folded tissue were generally excluded from the analysis. To calculate a colocalization channel between HS and IFNγ–GFP or IFNγ^ΔKRKR^–GFP, CD146 staining was used to calculate a mask, which was applied to the two channels of interest. By working with masked channels (HS in the basement membrane and GFP), all voxels outside of the CD146 region were set to 0 in these regions and thus excluded from building a colocalization channel. The object-based volumes, which indicate overlapping regions of masked HS and GFP, were calculated from colocalization and were normalized to CD146 volumes to get final values. Segmentation, colocalization analysis and visualization were performed with Imaris version 9.7.2 (Bitplane, Oxford Instruments).

### Detection of IFNγ

Part of the tumor was used to extract IFNγ from the tumor tissue. Tumors were weighed and dissociated in 4 ml of RPMI containing 300 µg ml^–1^ DNase I (Roche) using a C-tube and a GentleMACS dissociator (Miltenyi). C-tubes were centrifuged at 800*g* for 10 min, and supernatants were collected. Supernatants were centrifuged at 13,000*g* for 10 min, collected and stored at –80 °C for analysis. Sera and tumor supernatant were diluted 1:200 and 1:1,000, respectively, and IFNγ concentrations were determined using a mouse IFNγ ELISA (BD Biosciences).

### Serum half-life of IFNγ variants

To determine the serum half-life of IFNγ variants, 14- to 30-week-old *Ifng*^–/–^*Ifngr1*^–/–^ mice were injected with MCA313 cells, as described above. Mice received Dox for 3 d when tumors reached approximately 300 mm^3^, then Dox was removed. Serum was collected, and IFNγ concentrations were determined by ELISA. Half-life was calculated using a non-linear fit of a one-phase decay model in GraphPad Prism 9.

### Local release of IFNγ variants in vivo

Male or female C57BL/6 or Rag2-deficient mice were injected subcutaneously with 1 × 10^6^ MCA313^IFNγ-IND^, MCA313^IFNγΔKRKR-IND^, MCA313^IFNγ–GFP-IND^ or MCA313^IFNγΔKRKR–GFP-IND^ cancer cells into the left or right flank at 7–33 weeks of age (average of 16 weeks). Tumor size was assessed and calculated as described above. Administration of Dox via the drinking water was initiated when tumors reached 500–600 mm^3^. Animal well-being was monitored daily, and weight was determined beginning with Dox administration. In one cohort, temperature on the abdomen was assessed using an infrared thermometer for rodents (Bioseb). Sera and tumor supernatants were collected as described above.

### Generation of IFNγ^ΔKRKR^ mice using CRISPR–Cas9 gene editing

Knock-in mice lacking the EBD of IFNγ (IFNγ^ΔKRKR^) were generated using CRISPR–Cas9. gRNAs within the fourth exon of *Ifng* (chromosome 10, NCBI sequence NC_000076.6) were identified using the CRISPOR tool (http://crispor.tefor.net/)^36^. Two gRNAs (gRNA 1, 5′-ccagcctcaggaagcggaaa-3′; gRNA 2, 5′-cggaatccagcctcaggaag-3′) were chosen for targeting the KRKR sequence (agg and cgg). A 120-nucleotide (nt) repair template introducing the targeted 12-bp deletion was provided for homology-directed repair, spanning 60 nt upstream and downstream of the KRKR coding sequence (5′- caagcattcaatgagctcatccgagtggtccaccagctgttgccggaatccagcctcaggagtcgctgctgattcggggtggggaagagattgtcccaataagaataattctgccagcac-3′). Repair template and gRNAs (synthesized by IDT) were electroporated into C57BL/6N zygotes together with Cas9 protein using a Bio-Rad XCell electroporator, as described previously^37^. All offspring mice were genotyped by DNA isolation and PCR (5′-TCCATCTTCACTGACCATGATGT-3′ and 5′-CCAGATACAACCCCGCAATC-3′, 480 nt), followed by digestion with AciI (New England Biolabs) overnight. This allowed for discrimination between heterozygous and homozygous offspring.

### Characterization of IFNγ^ΔKRKR^ mouse lines

Homozygous IFNγ^ΔKRKR^, WT littermates and C57BL/6N (both male and female) mice were characterized at 13–16 weeks of age. Mice were killed, and blood was collected in potassium-EDTA tubes (Sarstedt). Peripheral blood mononuclear cells and splenocytes were stimulated for 24 h with anti-CD3/CD28 Dynabeads (Gibco), and supernatants were frozen at –80 °C until analysis by ELISA. Heart, liver, kidney, lung, spleen, brain, thymus and intestinal tissue were collected, fixed in formalin and embedded in paraffin. Tissue sections were stained with hematoxylin and eosin. Splenocytes were analyzed by flow cytometry.

### Induction of transient IFNγ responses

For anti-CD3 stimulation, mice received either 20 µg of anti-CD3 (clone 145-2C11) or the Armenian hamster IgG1, κ isotype (both ultra leaf-purified, Biolegend), in 200 µl of sterile PBS (Gibco) intravenously. LPS (Sigma) was injected intraperitoneally at 5 µg per g body weight in PBS (Gibco) or with PBS alone. Blood was obtained, and body weight and temperature were determined. Serum was collected as described above and stored at –80 °C until analysis by IFNγ ELISA (BD Bioscience).

### ATT

IFNγ^ΔKRKR^ mice were bred to TCR-I mice (B6.Cg-Tg(TcraY1,TcrbY1) 416Tev/J, 005236). TCR-I mice are transgenic for a TCR specific for epitope I of SV40 large T. For ATT experiments, female *Rag1*^–/–^ mice were injected subcutaneously with 1 × 10^6^ 16.113 cells. When tumors reached 500–600 mm³ in size, mice were treated with 1 × 10^6^ to 2 × 10^6^ TCR-I IFNγ or IFNγ^ΔKRKR^ T cells that were collected from TCR-I/IFNγ^ΔKRKR^ or TCR-I/IFNγ^WT^ mice. During ATT, weight and well-being were monitored from the day of transfer. Levels of serum IFNγ were determined using a mouse IFNγ ELISA (BD Biosciences). T cell expansion was monitored using anti-CD3-APC (clone 145-2C11, Biolegend), anti-CD8-BV421 (clone 53-6.7, Biolegend) and anti-Vβ7-PE (clone TR310, BD Biosciences).

### LCMV experiments

Mouse experiments were approved by Regierungspräsidium Freiburg (G-15/168). Male IFNγ^ΔKRKR^ mice, WT control littermates and C57BL/6 mice (Janvier; maintained under SPF conditions) were infected intravenously with 1.5 × 10^3^ to 3.0 × 10^3^ plaque-forming units of LCMV-Docile. Mice were killed if they lost >25% of their body weight or if they showed apathy or neurological failures. From day 5 onward, mice were monitored daily for body weight. Endpoint analysis was done between days 10 and 13 after infection, and clinical and biochemical parameters, such as ear temperature (ThermoScan 6022, BRAUN), blood cell counts (Sysmex KX-21 hematology analyzer), GPT, LDH, triglycerides and ferritin (Roche Modular Analytics Evo), were assessed. IFNγ levels in sera were determined by ELISA (Biolegend). LCMV titers in organ homogenates were quantified using a focus-forming assay^38^. For flow cytometry, splenocytes were stained with antibodies for ≥30 min at 4 °C. GP33-specific and NP396-specific CD8^+^ T cells were detected with PE-labeled H-2D^b^ tetramers from the Tetramer Core Facility at Baylor College of Medicine. For detection of intracellular cytokines, 10^6^ lymphocytes were stimulated with 10^–7^ M GP33 or NP396 peptide for 4 h, followed by surface staining and intracellular staining for IFNγ using a Cytofix/Cytoperm kit (BD Bioscience). Analyses were performed using an LSR Fortessa cytometer (BD Biosciences) and FlowJo software v8.8.7/v10.

Histopathological analysis of mice infected with LCMV-Docile was performed at the endpoint (days 10–13 after infection), and organs (liver, lung, brain, spleen and kidney) were taken, fixed in 4% buffered formalin and embedded in paraffin. Sections were stained with hematoxylin and eosin and analyzed by a veterinary pathologist.

### Software

Data analysis and plotting was performed using Microsoft Excel 2016 and 2019 or GraphPad Prism 9. Statistics were calculated using GraphPad Prism 9 or R software (version 4.1.2). Flow cytometry data were acquired using BD Diva or MACSQuantify and analyzed using FlowJo 10. Sequence analysis and cloning procedures were planned in SnapGene 5. Figure layouts were designed using Adobe Illustrator 2021 and Adobe InDesign 2021 as well as Biorender.com.

### Reporting summary

Further information on research design is available in the Nature Portfolio Reporting Summary linked to this article.

## Online content

Any methods, additional references, Nature Portfolio reporting summaries, source data, extended data, supplementary information, acknowledgements, peer review information; details of author contributions and competing interests; and statements of data and code availability are available at 10.1038/s41590-023-01420-5.

### Supplementary information

Supplementary Information Supplementary Tables 1 and 2.

Reporting Summary

Supplementary Video 1 Sixteen-micron sections of PFA-fixed and cryopreserved tumor tissue were stained for CD146 (yellow) and HS (red). Hoechst served as a counterstain for nuclei. The first part shows staining throughout a 10-µm volume. The second part (white) shows the calculated colocalization between IFNγ–GFP and HS within the CD146 channel. Sixteen-micron sections of PFA-fixed and cryopreserved tumor tissue were stained for CD146 (yellow) and HS (red). Hoechst served as a counterstain for nuclei. The first part shows staining throughout a 10-µm volume. The second part (white) shows the calculated colocalization between IFNγ–GFP and HS within the CD146 channel. Sixteen-micron sections of PFA-fixed and cryopreserved tumor tissue was stained for CD146 (yellow) and HS (red). Hoechst served as a counterstain for nuclei. The first part shows staining throughout a 10-µm volume. Cells have ceased to produce IFNγ–GFP 48 h after Dox treatment. Only singular cells still express IFNγ–GFP. The second part (white) shows the calculated colocalization between IFNγ–GFP and HS within the CD146 channel. Colocalized voxels are scattered across the tissue. Sixteen-micron sections of PFA-fixed and cryopreserved tumor tissue were stained for CD146 (yellow) and HS (red). Hoechst served as a counterstain for nuclei. The first part shows staining throughout a 10-µm volume. Cells have ceased to produce IFNγ–GFP 48 h after Dox treatment. Only singular cells still express IFNγ–GFP. The second part (white) shows the calculated colocalization between IFNγ–GFP and HS within the CD146 channel. Very few colocalization events can be observed.

### Source data

Source Data Fig. 1 Source data.

Source Data Fig. 2 Source data.

Source Data Fig. 3 Source data.

Source Data Fig. 4 Source data.

Source Data Extended Data Fig. 2 Source data.

Source Data Extended Data Fig. 4 Source data.

## Figures and Tables

**Extended Data Table 1:**
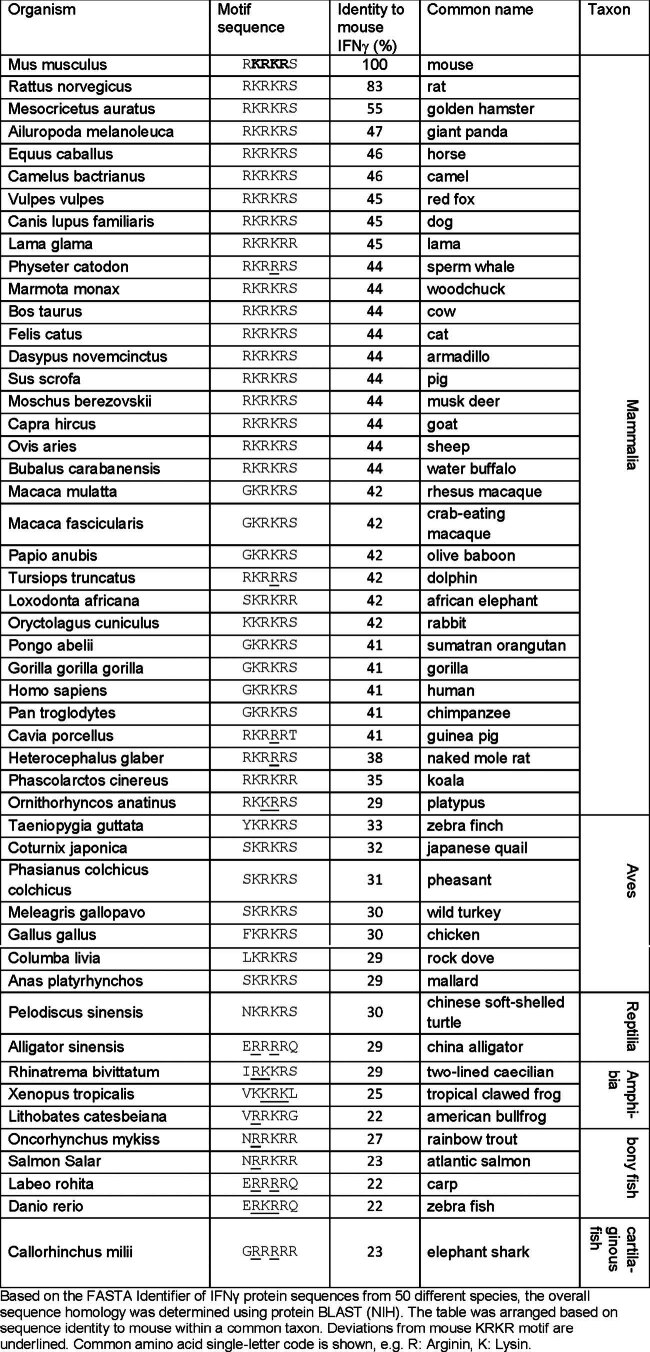
Sequence comparison of vertebrate IFNγ

Based on the FASTA Identifier of IFNγ protein sequences from 50 different species, the overall sequence homology was determined using protein BLAST (NIH). The table was arranged based on sequence identity to mouse within a common taxon. Deviations from mouse KRKR motif are underlined. Common amino acid single-letter code is shown, for example R: Arginin, K: Lysin.

## Data Availability

All data generated during this study are available in the article and supplementary files or from the corresponding author upon reasonable request. Source data are provided with this paper.
