# Publisher Correction: IFNγ binding to extracellular matrix prevents fatal systemic toxicity

**DOI:** 10.1038/s41590-023-01464-7

**Published:** 2023-02-22

**Authors:** Josephine Kemna, Evelyne Gout, Leon Daniau, Jessica Lao, Kristoffer Weißert, Sandra Ammann, Ralf Kühn, Matthias Richter, Christine Molenda, Anje Sporbert, Dario Zocholl, Robert Klopfleisch, Hugues Lortat-Jacob, Peter Aichele, Thomas Kammertoens, Thomas Blankenstein

**Affiliations:** 1 Max-Delbrück Center for Molecular Medicine in the Helmholtz Association, Molecular Immunology and Gene Therapy, Berlin, Germany; 2 Institut de Biologie Structurale, UMR 5075, University Grenoble Alpes, Centre National de la Recherche Scientifique, Commissariat à l’Énergie Atomique et aux Énergies Alternatives, Grenoble, France; 3 Institute for Immunodeficiency, Medical Center—University of Freiburg, Faculty of Medicine, University of Freiburg, Freiburg, Germany; 4 Faculty of Biology, Albert-Ludwigs-University of Freiburg, Freiburg, Germany; 5 Center for Chronic Immunodeficiency (CCI), Medical Center—University of Freiburg, Faculty of Medicine, University of Freiburg, Freiburg, Germany; 6 Transgenic Core Facility, Max-Delbrück Center for Molecular Medicine in the Helmholtz Association, Berlin, Germany; 7 Advanced Light Microscopy Core Facility, Max-Delbrück Center for Molecular Medicine in the Helmholtz Association, Berlin, Germany; 8 Charité—Universitätsmedizin Berlin, Corporate Member of Freie Universität Berlin and Humboldt-Universität zu Berlin, Institute of Biometry and Clinical Epidemiology, Berlin, Germany; 9 Department of Veterinary Medicine, Institute of Veterinary Pathology, Freie Universität Berlin, Berlin, Germany; 10 Institute of Immunology, Charité Unversitätsmedizin, Campus Buch, Berlin, Germany

**Keywords:** Interferons, Chronic inflammation

Correction to: *Nature Immunology* 10.1038/s41590-023-01420-5. Published online 2 February 2023.

In the version of this article originally published, there was an error in the order of magnitude for the units displayed on the *y* axes of the Figure 4g HBG (g/dl) panel and Figure 4h Triglycerides (mg/dl) panel, where the units now reading “20, 15, 10, 5, 0” and “200, 150, 100, 50, 0” appeared in error as “2,000, 1,500, 1,000, 500, 0” and “20,000, 15,000, 10,000, 5,000, 0,” respectively. The units have been corrected in the HTML and PDF versions of the article.

