# Author Correction: IFNγ binding to extracellular matrix prevents fatal systemic toxicity

**DOI:** 10.1038/s41590-024-01835-8

**Published:** 2024-04-19

**Authors:** Josephine Kemna, Evelyne Gout, Leon Daniau, Jessica Lao, Kristoffer Weißert, Sandra Ammann, Ralf Kühn, Matthias Richter, Christine Molenda, Anje Sporbert, Dario Zocholl, Robert Klopfleisch, Anja Schütz, Hugues Lortat-Jacob, Peter Aichele, Thomas Kammertoens, Thomas Blankenstein

**Affiliations:** 1 Max-Delbrück Center for Molecular Medicine in the Helmholtz Association, Molecular Immunology and Gene Therapy, Berlin, Germany; 2 Institut de Biologie Structurale, UMR 5075, University Grenoble Alpes, Centre National de la Recherche Scientifique, Commissariat à l’Énergie Atomique et aux Énergies Alternatives, Grenoble, France; 3 Institute for Immunodeficiency, Medical Center—University of Freiburg, Faculty of Medicine, University of Freiburg, Freiburg, Germany; 4 Faculty of Biology, Albert-Ludwigs-University of Freiburg, Freiburg, Germany; 5 Center for Chronic Immunodeficiency (CCI), Medical Center—University of Freiburg, Faculty of Medicine, University of Freiburg, Freiburg, Germany; 6 Transgenic Core Facility, Max-Delbrück Center for Molecular Medicine in the Helmholtz Association, Berlin, Germany; 7 Advanced Light Microscopy Core Facility, Max-Delbrück Center for Molecular Medicine in the Helmholtz Association, Berlin, Germany; 8 Charité—Universitätsmedizin Berlin, Corporate Member of Freie Universität Berlin and Humboldt-Universität zu Berlin, Institute of Biometry and Clinical Epidemiology, Berlin, Germany; 9 Department of Veterinary Medicine, Institute of Veterinary Pathology, Freie Universität Berlin, Berlin, Germany; 10 Protein Production & Characterization Core Facility, Max-Delbrück Center for Molecular Medicine in the Helmholtz Association, Berlin, Germany; 11 Institute of Immunology, Charité Unversitätsmedizin, Campus Buch, Berlin, Germany

**Keywords:** Interferons, Chronic inflammation

Correction to: *Nature Immunology* 10.1038/s41590-023-01420-5, published online 2 February 2023.

In the version of the article initially published, Anja Schütz (Protein Production & Characterization Core Facility, Max-Delbrück Center for Molecular Medicine in the Helmholtz Association, Berlin, Germany), who produced the recombinant proteins used in this study, was inadvertently omitted from the author list. This has now been corrected in the PDF and HTML versions of the article.

