# Supplementary material for: IFNγ binding to extracellular matrix prevents fatal systemic toxicity
Source: Nat Immunol. 2023 Feb 2;24(3):414–22. doi: 10.1038/s41590-023-01420-5 (PMC9977683; doi:10.1038/s41590-023-01420-5)
Supplement: Supplementary file 1 — Supplementary Tables 1 and 2. [file 41590_2023_1420_MOESM1_ESM.pdf]

nature immunology

Article https://doi.org/10.1038/s41590-023-01420-5

IFNγ binding to extracellular matrix prevents fatal systemic toxicity

In the format provided by the authors and unedited

FASTA identifiers of vertebrate species for sequence analysis

<table>
<tr><th>Species</th><th>Common name</th><th>Taxonomic identifier (NCBI)</th><th>IFNg FASTA identifier</th><th>Taxon</th></tr>
<tr><td>Mus musculus</td><td>mouse</td><td>10090</td><td>>sp|P01580|IFNG_MOUSE Interferon gamma OS=Mus musculus OX=10090 GN=Ifng PE=1 SV=1</td><td></td></tr>
<tr><td>Rattus norvegicus</td><td>rat</td><td>10116</td><td>>sp|P01581|IFNG_RAT Interferon gamma OS=Rattus norvegicus OX=10116 GN=Ifng PE=2 SV=1</td><td></td></tr>
<tr><td>Mesocricetus auratus</td><td>golden hamster</td><td>10036</td><td>>sp|O35497|IFNG_MESAU Interferon gamma OS=Mesocricetus auratus OX=10036 GN=IFNG PE=2 SV=1</td><td></td></tr>
<tr><td>Ailuropoda melanoleuca</td><td>giant panda</td><td>9646</td><td>>tr|C8CBV8|C8CBV8_AILME Interferon gamma OS=Ailuropoda melanoleuca OX=9646 GN=IFNG PE=2 SV=1</td><td></td></tr>
<tr><td>Equus caballus</td><td>horse</td><td>9796</td><td>>sp|P42160|IFNG_HORSE Interferon gamma OS=Equus caballus OX=9796 GN=IFNG PE=2 SV=1</td><td></td></tr>
<tr><td>Camelus bactrianus</td><td>camel</td><td>9837</td><td>>sp|Q865W6|IFNG_CAMBA Interferon gamma OS=Camelus bactrianus OX=9837 GN=IFNG PE=2 SV=1</td><td></td></tr>
<tr><td>Vulpes vulpes</td><td>red fox</td><td>9627</td><td>>sp|Q25BC0|IFNG_VULVU Interferon gamma OS=Vulpes vulpes OX=9627 GN=IFNG PE=2 SV=1</td><td></td></tr>
<tr><td>Canis lupus familiaris</td><td>dog</td><td>9615</td><td>>sp|P42161|IFNG_CANLF Interferon gamma OS=Canis lupus familiaris OX=9615 GN=IFNG PE=2 SV=2</td><td></td></tr>
<tr><td>Lama glama</td><td>lama</td><td>9844</td><td>>sp|Q865X1|IFNG_LAMGL Interferon gamma OS=Lama glama OX=9844 GN=IFNG PE=2 SV=1</td><td></td></tr>
<tr><td>Physeter catodon</td><td>sperm whale</td><td>9755</td><td>>tr|A0A2Y9EPI7|A0A2Y9EPI7_PHYCD Interferon gamma OS=Physeter catodon OX=9755 GN=IFNG PE=3 SV=1</td><td></td></tr>
<tr><td>Marmota monax</td><td>woodchuck</td><td>9995</td><td>>sp|O35735|IFNG_MARMO Interferon gamma OS=Marmota monax OX=9995 GN=IFNG PE=2 SV=2</td><td></td></tr>
<tr><td>Bos taurus</td><td>cow</td><td>9913</td><td>>sp|P07353|IFNG_BOVIN Interferon gamma OS=Bos taurus OX=9913 GN=IFNG PE=1 SV=1</td><td></td></tr>
<tr><td>Felis catus</td><td>cat</td><td>9685</td><td>>sp|P46402|IFNG_FELCA Interferon gamma OS=Felis catus OX=9685 GN=IFNG PE=2 SV=1</td><td></td></tr>
</table>

Dasypus novemcinctus	armadillo	9361	>sp|Q1WM28|IFNG_DASNO Interferon gamma OS=Dasypus novemcinctus OX=9361 GN=IFNG PE=2 SV=1	Mammalia
Sus scrofa	pig	9823	>sp|P17803|IFNG_PIG Interferon gamma OS=Sus scrofa OX=9823 GN=IFNG PE=2 SV=1	
Moschus berezovskii	musk deer	68408	>sp|Q647G2|IFNG_MOSBE Interferon gamma OS=Moschus berezovskii OX=68408 GN=IFNG PE=2 SV=1	
Capra hircus	goat	9925	>tr|A3FG73|A3FG73_CAPHI Interferon gamma OS=Capra hircus OX=9925 GN=IFNG PE=2 SV=1	
Ovis aries	sheep	9940	>sp|P17773|IFNG_SHEEP Interferon gamma OS=Ovis aries OX=9940 GN=IFNG PE=2 SV=1	
Bubalus carabanensis	water buffalo	346063	>sp|Q2PE75|IFNG_BUBCA Interferon gamma OS=Bubalus carabanensis OX=346063 GN=IFNG PE=2 SV=1	
Macaca mulatta	rhesus macaque	9544	>sp|P63310|IFNG_MACMU Interferon gamma OS=Macaca mulatta OX=9544 GN=IFNG PE=2 SV=1	
Macaca fascicularis	crab-eating macaque	9541	>sp|P63309|IFNG_MACFA Interferon gamma OS=Macaca fascicularis OX=9541 GN=IFNG PE=2 SV=1	
Papio anubis	olive baboon	9555	>sp|Q865Y4|IFNG_PAPAN Interferon gamma OS=Papio anubis OX=9555 GN=IFNG PE=2 SV=1	
Tursiops truncatus	dolphin	9739	>sp|Q9TV67|IFNG_TURTR Interferon gamma OS=Tursiops truncatus OX=9739 GN=IFNG PE=2 SV=1	
Loxodonta africana	african elephant	9785	>tr|G3SUC6|G3SUC6_LOXAF Interferon gamma OS=Loxodonta africana OX=9785 GN=IFNG PE=3 SV=1	
Oryctolagus cuniculus	rabbit	9986	>sp|P30123|IFNG_RABIT Interferon gamma OS=Oryctolagus cuniculus OX=9986 GN=IFNG PE=1 SV=1	
Pongo abelii	sumatran orangutan	9601	>tr|H2NHZ4|H2NHZ4_PONAB Interferon gamma OS=Pongo abelii OX=9601 GN=IFNG PE=3 SV=1	
Gorilla gorilla gorilla	gorilla	9595	>tr|G3QT96|G3QT96_GORGO Interferon gamma OS=Gorilla gorilla gorilla OX=9595 PE=3 SV=1	
Homo sapiens	human	9606	>sp|P01579|IFNG_HUMAN Interferon gamma OS=Homo sapiens OX=9606 GN=IFNG PE=1 SV=1	
Pan troglodytes	chimpanzee	9598	>tr|H2Q6F3|H2Q6F3_PANTR Interferon gamma OS=Pan troglodytes OX=9598 GN=IFNG PE=3 SV=1	

<table>
<tr><td>Cavia porcellus</td><td>guinea pig</td><td>10141</td><td>>tr|Q8CGS0|Q8CGS0_CAVPO Interferon gamma OS=Cavia porcellus OX=10141 GN=Ifng PE=2 SV=1</td><td></td></tr>
<tr><td>Heterocephalus glaber</td><td>naked mole rat</td><td>10181</td><td>>tr|G5BGB5|G5BGB5_HETGA Interferon gamma OS=Heterocephalus glaber OX=10181 GN=GW7_16003 PE=3 SV=1</td><td></td></tr>
<tr><td>Phascolarctos cinereus</td><td>koala</td><td>38626</td><td>>tr|S5SA69|S5SA69_PHACI Interferon gamma OS=Phascolarctos cinereus OX=38626 GN=IFNgamma PE=2 SV=1</td><td></td></tr>
<tr><td>Ornithorhyncos anatinus</td><td>platypus</td><td>9258</td><td>>tr|F6PQX2|F6PQX2_ORNAN Interferon gamma OS=Ornithorhynchus anatinus OX=9258 GN=IFNG PE=3 SV=1</td><td></td></tr>
<tr><td>Taeniopygia guttata</td><td>zebra finch</td><td>59729</td><td>>tr|H0Z8K7|H0Z8K7_TAEGU Interferon gamma OS=Taeniopygia guttata OX=59729 GN=IFNG PE=3 SV=1</td><td></td></tr>
<tr><td>Coturnix japonica</td><td>japanese quail</td><td>93934</td><td>>sp|O57571|IFNG_COTJA Interferon gamma OS=Coturnix japonica OX=93934 GN=IFNG PE=3 SV=1</td><td></td></tr>
<tr><td>Phasianus colchicus colchicus</td><td>pheasant</td><td>9057</td><td>>sp|O57608|IFNG_PHACO Interferon gamma OS=Phasianus colchicus colchicus OX=9057 GN=IFNG PE=3 SV=1</td><td></td></tr>
<tr><td>Meleagris gallopavo</td><td>wild turkey</td><td>9103</td><td>>tr|G1NDA2|G1NDA2_MELGA Interferon gamma OS=Meleagris gallopavo OX=9103 GN=IFNG PE=3 SV=1</td><td>Aves</td></tr>
<tr><td>Gallus gallus</td><td>chicken</td><td>9031</td><td>>sp|P49708|IFNG_CHICK Interferon gamma OS=Gallus gallus OX=9031 GN=IFNG PE=2 SV=1</td><td></td></tr>
<tr><td>Columba livia</td><td>rock dove</td><td>8932</td><td>>tr|A0A2I0MJ31|A0A2I0MJ31_COLLI Interferon gamma OS=Columba livia OX=8932 GN=IFNG PE=3 SV=1</td><td></td></tr>
<tr><td>Anas platyrhynchos</td><td>mallard</td><td>8839</td><td>>sp|Q9YGB9|IFNG_ANAPL Interferon gamma OS=Anas platyrhynchos OX=8839 GN=IFNG PE=2 SV=1</td><td></td></tr>
<tr><td>Pelodiscus sinensis</td><td>chinese soft-shelled turtle</td><td>13735</td><td>>tr|K7GF20|K7GF20_PELSI Interferon gamma OS=Pelodiscus sinensis OX=13735 GN=IFN-gamma PE=2 SV=1</td><td>Reptilia</td></tr>
<tr><td>Alligator sinensis</td><td>china alligator</td><td>38654</td><td>>tr|A0A1U7RPI8|A0A1U7RPI8_ALLSI Interferon gamma OS=Alligator sinensis OX=38654 GN=LOC102384045 PE=3 SV=1</td><td></td></tr>
<tr><td>Rhinatrema bivittatum</td><td>two-lined caecilian</td><td>194408</td><td>>XP_029453489.1 interferon gamma [Rhinatrema bivittatum]</td><td></td></tr>
</table>

Xenopus tropicalis	tropical clawed frog	8364	>tr|B6RCW1|B6RCW1_XENTR Interferon gamma (Fragment) OS=Xenopus tropicalis OX=8364 GN=ifng PE=2 SV=1	Amphibia
Lithobates catesbeiana	american bullfrog	8400	>tr|A0A2G9S3S3|A0A2G9S3S3_LITCT Interferon gamma OS=Lithobates catesbeiana OX=8400 GN=AB205_0117080 PE=4 SV=1	
Oncorhynchus mykiss	rainbow trout	8022	>tr|Q5QSL2|Q5QSL2_ONCMY Interferon gamma OS=Oncorhynchus mykiss OX=8022 GN=ifng PE=2 SV=1	bony fish
Salmon Salar	atlantic salmon	8030	>tr|Q5QRA1|Q5QRA1_SALSA Interferon gamma OS=Salmo salar OX=8030 GN=ifng PE=2 SV=1	
Labeo rohita	carp	84645	>tr|F1D7Y1|F1D7Y1_LABRO Interferon gamma OS=Labeo rohita OX=84645 PE=2 SV=1	
Danio rerio	zebra fish	7955	>tr|Q5RHV5|Q5RHV5_DANRE Interferon gamma 1 OS=Danio rerio OX=7955 GN=ifng1 PE=4 SV=1	
Callorhinchus milii	elephant shark	7868	>XP_007903185.1 PREDICTED: interferon gamma [Callorhinchus milii]	cartilaginous fish

**Amino acid sequences of proteins used for SPR**
**IFNγ:**
SHGTVIESLESLNNYFNSSGIDVEEKSLFLDIWRNWQKDGDMKILQSQIISFYLRLFEVLKDNQAISNNISVIESHLITTFFSNSKA<br>KKDAFMSIAKFEVNNPQVQRQAFNELIRVVHQLLPESSLRKRKRSRC
**IFNγΔKRKR:**<br>SHGTVIESLESLNNYFNSSGIDVEEKSLFLDIWRNWQKDGDMKILQSQIISFYLRLFEVLKDNQAISNNISVIESHLITTFFSNSKA<br>KKDAFMSIAKFEVNNPQVQRQAFNELIRVVHQLLPESSLRSRC
**IFNγ-GFP**:<br>SHGTVIESLESLNNYFNSSGIDVEEKSLFLDIWRNWQKDGDMKILQSQIISFYLRLFEVLKDNQAISNNISVIESHLITTFFSNSKA<br>KKDAFMSIAKFEVNNPQVQRQAFNELIRVVHQLLPESSLRKRKRSRCGGGGSGGGGSGGGGSMVSKGEELFTGVVPILVELD<br>GDVNGHKFSVSGEGEGDATYGKLTLKFICTTGKLPVPWPTLVTTLTYGVQCFSRYPDHMKQHDFFKSAMPEGYVQERTIFFK<br>DDGNYKTRAEVKFEGDTLVNRIELKGIDFKEDGNILGHKLEYNYNSHNVYIMADKQKNGIKVNFKIRHNIEDGSVQLADHYQ<br>QNTPIGDGPVLLPDNHYLSTQSALSKDPNEKRDHMVLLEFVTAAGITLGMDELYK
**IFNγΔKRKR-GFP:**<br>SHGTVIESLESLNNYFNSSGIDVEEKSLFLDIWRNWQKDGDMKILQSQIISFYLRLFEVLKDNQAISNNISVIESHLITTFFSNSKA<br>KKDAFMSIAKFEVNNPQVQRQAFNELIRVVHQLLPESSLRSRCGGGGSGGGGSGGGGSMVSKGEELFTGVVPILVELDGDVN<br>GHKFSVSGEGEGDATYGKLTLKFICTTGKLPVPWPTLVTTLTYGVQCFSRYPDHMKQHDFFKSAMPEGYVQERTIFFKDDGN<br>YKTRAEVKFEGDTLVNRIELKGIDFKEDGNILGHKLEYNYNSHNVYIMADKQKNGIKVNFKIRHNIEDGSVQLADHYQQNTPI<br>GDGPVLLPDNHYLSTQSALSKDPNEKRDHMVLLEFVTAAGITLGMDELYK
**IFNγR1:**<br>GSALTSTEDPEPPSVPVPTNVLIKSYNLNPVVCWEYQNMSQTPIFTVQVKVYSGSWTDSCTNISDHCCNIYEQIMYPDVSAWA<br>RVKAKVGQKESDYARSKEFLMCLKGKVGPPGLEIRRKKEEQLSVLVFHPEVVVNGESQGTMFGDGSTCYTFDYTVYVEHNR<br>SGEILHTKHTVEKEECNETLCELNISVSTLDSRYCISVDGISSFWQVRTEKSKDVCIPPFHDDRKDSENLYFQGLEHHHHHH
