# Supplementary material for: IFNγ binding to extracellular matrix prevents fatal systemic toxicity
Source: Nat Immunol. 2023 Feb 2;24(3):414–22. doi: 10.1038/s41590-023-01420-5 (PMC9977683; doi:10.1038/s41590-023-01420-5)
Supplement: Supplementary file 2 — Reporting Summary [file 41590_2023_1420_MOESM2_ESM.pdf]

nature portfolio

Corresponding author(s): Thomas Blankenstein

Last updated by author(s): Nov 16, 2022

Reporting Summary

Nature Portfolio wishes to improve the reproducibility of the work that we publish. This form provides structure for consistency and transparency in reporting. For further information on Nature Portfolio policies, see our Editorial Policies and the Editorial Policy Checklist.

Statistics

For all statistical analyses, confirm that the following items are present in the figure legend, table legend, main text, or Methods section.

n/a	Confirmed	
☐	☒	The exact sample size (*n*) for each experimental group/condition, given as a discrete number and unit of measurement
☐	☒	A statement on whether measurements were taken from distinct samples or whether the same sample was measured repeatedly
☐	☒	The statistical test(s) used AND whether they are one- or two-sided<br>*Only common tests should be described solely by name; describe more complex techniques in the Methods section.*
☒	☐	A description of all covariates tested
☒	☐	A description of any assumptions or corrections, such as tests of normality and adjustment for multiple comparisons
☐	☒	A full description of the statistical parameters including central tendency (e.g. means) or other basic estimates (e.g. regression coefficient) AND variation (e.g. standard deviation) or associated estimates of uncertainty (e.g. confidence intervals)
☐	☒	For null hypothesis testing, the test statistic (e.g. *F*, *t*, *r*) with confidence intervals, effect sizes, degrees of freedom and *P* value noted<br>*Give P values as exact values whenever suitable.*
☒	☐	For Bayesian analysis, information on the choice of priors and Markov chain Monte Carlo settings
☒	☐	For hierarchical and complex designs, identification of the appropriate level for tests and full reporting of outcomes
☒	☐	Estimates of effect sizes (e.g. Cohen's *d*, Pearson's *r*), indicating how they were calculated

Our web collection on statistics for biologists contains articles on many of the points above.

Software and code

Policy information about availability of computer code

Data collection	Microsoft Excel 2016 and 2019, Miltenyi: MacsQuantify 2.11, BD FACS Calibur Software, BD FACS Diva Software 9.0.2, Biaevaluation Software 3.1., Imaris 9.7.2, Quicklink Revelation Software 4.25, Image Lab
Data analysis	Microsoft Excel 2019, GraphPad Prism 9, FlowJo 10, Imaris 9.7.2, Image Lab, Snap Gene 5.1.7, R 4.1.2

For manuscripts utilizing custom algorithms or software that are central to the research but not yet described in published literature, software must be made available to editors and reviewers. We strongly encourage code deposition in a community repository (e.g. GitHub). See the Nature Portfolio guidelines for submitting code & software for further information.

Data

Policy information about availability of data

All manuscripts must include a data availability statement. This statement should provide the following information, where applicable:

- Accession codes, unique identifiers, or web links for publicly available datasets
- A description of any restrictions on data availability
- For clinical datasets or third party data, please ensure that the statement adheres to our policy

Fasta Identifiers of analysed IFNg sequences from different species can be found in supplementary information. Data from central experiments showing that IFNγ binding to extracellular matrix prevents fatal systemic toxicity are provided as excel sheets in the supplementary information. We have provided a data availability statement.

Field-specific reporting

Please select the one below that is the best fit for your research. If you are not sure, read the appropriate sections before making your selection.

☒ Life sciences ☐ Behavioural & social sciences ☐ Ecological, evolutionary & environmental sciences

For a reference copy of the document with all sections, see nature.com/documents/nr-reporting-summary-flat.pdf

Life sciences study design

All studies must disclose on these points even when the disclosure is negative.

Sample size	Sample Size for animal experiment was determined based on the statistical calculations during the application for the respective license. Numbers of animals in the respective experimental group were then typically split into at least two independent experiments (biological replicates).
Data exclusions	No data was excluded.
Replication	Experiments were replicated at least twice if not stated otherwise.
Randomization	Mice were randomly assigned into groups when injected with tumor cells. For ATT experiments, mice were allocated to groups based on equal distribution in tumor size between different groups.
Blinding	Investigators were not blinded as endpoint criteria of mouse experiments were defined prior to experiments. Mice reaching endpoint criteria were analysed independently of their group allocation.

Reporting for specific materials, systems and methods

We require information from authors about some types of materials, experimental systems and methods used in many studies. Here, indicate whether each material, system or method listed is relevant to your study. If you are not sure if a list item applies to your research, read the appropriate section before selecting a response.

Materials & experimental systems

n/a	Involved in the study	
☐	☒	Antibodies
☐	☒	Eukaryotic cell lines
☒	☐	Palaeontology and archaeology
☐	☒	Animals and other organisms
☒	☐	Human research participants
☒	☐	Clinical data
☒	☐	Dual use research of concern

Methods

n/a	Involved in the study	
☒	☐	ChIP-seq
☐	☒	Flow cytometry
☒	☐	MRI-based neuroimaging

Antibodies

Antibodies used

CD45.2 APC, 104, Biolegend 109814, Mouse IgG2a, k (1:100)
CD3 FITC, 145-2C11, BD Biosciences 553062, Armenian Hamster IgG1, k (1:20)
CD45.2 BV421, 104, Biolegend 109832, Mouse IgG2a, k (1:100)
CD19 FITC, 6D5, Biolegend 115506, Rat IgG2a, k (1:100)
CD11b BV510, M1/70, Biolegend 101263, Rat IgG2b, k (1:100)
CD11c FITC, HL3, BD Biosciences 553801, Hamster IgG1 (1:50)
NK1.1 APC, Miltenyi Biotech 130-102-350, (1:20)
RGS·His Antibody, 34650, Quiagen
H-2Kb/H-2Db-Biotin, 28-8-6, BD Biosciences 553575, (1:100)
Streptavidin-APC, BD Biosciences 554067, (1:200)
Heparan sulfate proteoglycan 2 antibody, A7L6, Abcam ab2501, (1:100)
goat anti-rat-Alexa568, Invitrogen A11077, (1:250)
CD146-Alexa647, ME-9F1, Biolegend 134702, (1:33)
Hoechst 33342, Sigma Aldrich
ultra-leaf-purified CD3, 145-2C11, Biolegend 100340, Armenian Hamster IgG1, k, (n.a.)
ultra-leaf purified Armenian Hamster IgG1, k isotype control, Biolegend 400940 (n.a.)
CD8 BV421, 53-6.7, Biolegend 100753, (1:50-1:100)
Vb7 PE, TR310, BD Biosciences 553216 (1:50)
CD3-APC, 145-2C11, Biolegend 100312 (1:50)
Surface staining LCMV Panel
CD4-APC/Fire750, RM4-4, Biolegend 116019, Rat IgG2b, k

CD8-BV510 53-6.7, Biolegend 100751, Rat IgG2a, k
KLRG-1-PerCP-Cy5.5, 2F1, BD 563595, Syrian hamster IgG
CD127-BV421, A7R34, Biolegend 135023, Rat IgG2a, k
CD44-APC, IM7, eBioscience 17-0441, Rat IgG2b, k
CD62L-BV650, MEL-14, Biolegend 104453, Rat IgG2a, k
PD-1-BV785, 29F.1A12, Biolegend 135225, Rat IgG2a, k
LAG3-PE-Cy7, C9B7W, Biolegend 125208, Rat IgG1, k
Tet-GP33-PE , Baylor College of Medicine
Tet-NP396-PE Baylor College of Medicine
CD45.2-BV711, 104, Biolegend 109847,Mouse (SJL) IgG2a, κ 1:125
CD19-PE, 6D5, Biolegend 115508,Rat IgG2a, κ 1:250
CD4-PE-cy7, RM4-5, Biolegend 116016, Rat IgG2a, κ 1:250
CD8-FITC, 53-6.7, Biolegend 100706, Rat IgG2a, κ 1:250
CD3-BV421, 145-2C11, Biolegend 100336, Armenian Hamster IgG 1:125
CD274 (PD-L1)-APC, 10F.9G2, Biolegend 124312, Rat IgG2b, κ 1:250
Fc block, 93, Biolegend 101302, Rat IgG2a, λ
CD4 Fitc RM4-5, Biolegend 100510, Rat IgG2a, κ 1:250
CD8 BV421 53-6.7, Biolegend Rat IgG2a, κ 1:250
CD19 PE 6D5, Biolegend 100738, Rat IgG2a, κ 1:250
Mouse anti-Stat1 PE 4a, BD Biosciences 612564, Mouse IgG2a 1:5
Isotype control PE MOPC-173, BD Biosciences 558595, Mouse IgG2a,k

Intracellular cytokine staining LCMV Panel
CD8-PerCP-Cy5.5, 53-6.7, Biolegend, Rat IgG2a, k
CD4-BV650, RM4-4, Biolegend, Rat IgG2b, k
IFNγ-APC, XMG1.2, Biolegend, Rat IgG1, k
TNF-FITC, MP6-XT22, Biolegend, Rat IgG1, k

Validation

Antibodies were validated using the respective antigen on cells or expressed by cells and controlled by respective isotypes. For heparan sulfate staining, stainings without the primary antibodies served as controls. The Perlecan - heparan sulfate antibody was previously reported for mouse tissue staining: Tsiantoulas, D., et al. (2021). "APRIL limits atherosclerosis by binding to heparan sulfate proteoglycans." Nature 597(7874): 92-+

Eukaryotic cell lines

Policy information about cell lines

Cell line source(s)

B16F10, MCA313, 16.113, HEK293T

Authentication

B16F10 cells were authenticated by IFNg-dependent upregulation of MHC I, as determined by flow cytometry, and pigmentation of the cells. MCA313 were identified based on their morphology (fibroblast-like) and by PCR specific for the neomycin resistance cassette within the inactivated IFNgR locus. In addition the expression of mouse MHC I was confirmed by flow cytometry. 16.113 cell line was identified based on IFNg secretion by TCR I - T cells (specific for the Large T antigen expressed by the tumor cells) upon co-culture. HEK293T were only used to produce retroviral vector particles and not in any experiments.

Mycoplasma contamination

All cell lines were routinely tested and were negative by PCR-Test for Mycoplasma.

Commonly misidentified lines
(See ICLAC register)

Name any commonly misidentified cell lines used in the study and provide a rationale for their use.

Animals and other organisms

Policy information about studies involving animals; ARRIVE guidelines recommended for reporting animal research

Laboratory animals

Rag1-/- (B6.129S7-Rag1tm1Mom/J, 002216), TCR-I transgenic mice (B6.Cg-Tg(TcraY1,TcrbY1)416Tev/J, 005236), C57BL/6N (005304), C57BL/6J (000664), B6.129S7-Ifng<tm1Ts>/J (002287) and B6.129S7-Ifngr1(tm1Agt)/J (003288) mouse strains were obtained from The Jackson Laboratory. For colocalisation experiments, male and female mice were used at 11-43 weeks of age. For local release of IFNg, male and female mice were used at 7-33 weeks of age. Female Rag1-/- mice were injected with cancer cells at 7-16 weeks of age. IFNg-delKRKR mice for LCMV Experiments were infected at 11-19 weeks. C57BL/6 mice from Janvier were aged and sex matched.
Arrive criteria were adhered to. 1.For each experiment the study design and controls are described. 2. Sample size was calculated beforehand with the application and if not indicated otherwise at least two biological replicates were performed to test reproducibility. 3. No animals were excluded. 4. Mice were randomly assigned into groups when injected with tumor cells. For ATT experiments, mice were allocated to groups based on equal distribution in tumor size between different groups.5. No blinding was performed. 6. All outcome measures (for statistical analysis) are defined. The hypothesis are stated. 7. Statistical methods are described in the Text and M+M section. 8. Appropriate information on mice (genotype, sex, age, provenience, health – and immune status) is given. 9. Experiments and 10. Results are described.

Wild animals

Provide details on animals observed in or captured in the field; report species, sex and age where possible. Describe how animals were caught and transported and what happened to captive animals after the study (if killed, explain why and describe method; if released, say where and when) OR state that the study did not involve wild animals.

Field-collected samples	*For laboratory work with field-collected samples, describe all relevant parameters such as housing, maintenance, temperature, photoperiod and end-of-experiment protocol OR state that the study did not involve samples collected from the field.*
Ethics oversight	Landesamt für Gesundheit und Soziales, Berlin (G-322/10, G-114/17, G-0058/16)<br>Regierungspräsidium Freiburg (G-15/168)

Note that full information on the approval of the study protocol must also be provided in the manuscript.

Flow Cytometry

Plots

Confirm that:

☒ The axis labels state the marker and fluorochrome used (e.g. CD4-FITC).

☒ The axis scales are clearly visible. Include numbers along axes only for bottom left plot of group (a 'group' is an analysis of identical markers).

☒ All plots are contour plots with outliers or pseudocolor plots.

☒ A numerical value for number of cells or percentage (with statistics) is provided.

Methodology

Sample preparation	Blood: whole blood was collected in K2-EDTA Tubes and subjected to Fc Block for 5-10 minutes, followed by 20 min incubation with antibody mixtures in PBS. ACK lysis was performed, samples were washed twice before acquisition.<br>Cancer cells from cell culture: Cells were detached using trypsin or a cell scraper and washed with PBS.<br>Splenocytes: Spleens were excised and single cells suspension were generated using a 40µm cell strainer. Suspensions were subjected to ACK Lysis. Stainings were performed similar to blood stainings.
Instrument	MACSQuant Analyzer, Milteny<br>Facs Calibur, BD Biosciences<br>Aria Fusion Cell Sorter, BD Biosciences<br>LSR Fortessa cytometer (BD Biosciences)
Software	FlowJo 8.8.7 & 10, BD Diva,
Cell population abundance	MCA313 cells were single cell sorted.
Gating strategy	Data involving cell lines were gated on FSC/SSC, followed by gating for the analysed markers. Boundaries between positive and negative populations were set based on FMO controls or cell lines negative for the specific markers.<br>Dead cells were excluded from analysis.<br>For characterization of splenocytes from IFNg-delKRKR mice, FCS/SSC was followed by CD45.2-positive selection and subsequent analysis of respective surface markers. Isotype controls were used to set boundaries.<br>Blood lymphocytes were gated based on FSC/SSC, followed by discrimination between CD3 negative and positive, based on isotype control. The CD3-positive fraction was then analysed for CD8 and Vb7 staining. For Vb7 boundaries, the isotype was used. In all cases, isotype controls were performed on the same samples as the primary staining.

☒ Tick this box to confirm that a figure exemplifying the gating strategy is provided in the Supplementary Information.
